# Aberrant PI3Kδ splice isoform as a potential biomarker and novel therapeutic target for endocrine cancers

**DOI:** 10.3389/fendo.2023.1190479

**Published:** 2023-08-21

**Authors:** Siyoung Ha, Himali Gujrati, Bi-Dar Wang

**Affiliations:** ^1^ Department of Pharmaceutical Sciences, School of Pharmacy and Health Professions, University of Maryland Eastern Shore, Princess Anne, MD, United States; ^2^ Hormone Related Cancers Program, University of Maryland Greenebaum Comprehensive Cancer Center, Baltimore, MD, United States

**Keywords:** aberrant PI3Kδ splice isoform, endocrine cancers, PTEN, precision prognostic biomarker, Idelalisib resistance, synergistic drug effect

## Abstract

**Introduction:**

PI3K/AKT signaling pathway is upregulated in a broad spectrum of cancers. Among the class I PI3Ks (PI3Kδ/β/δ isoforms), PI3Kδ has been implicated in hematologic cancers and solid tumors. Alternative splicing is a post-transcriptional process for acquiring proteomic diversity in eukaryotic cells. Emerging evidence has highlighted the involvement of aberrant mRNA splicing in cancer development/progression.

**Methods:**

Our previous studies revealed that *PIK3CD-S* is an oncogenic splice variant that promotes tumor aggressiveness and drug resistance in prostate cancer (PCa). To further evaluate the potential of utilizing PI3Kδ-S (encoded from *PIK3CD-S*) as a cancer biomarker and/or drug target, comprehensive analyses were performed in a series of patient samples and cell lines derived from endocrine/solid tumors. Specifically, IHC, immunofluorescence, western blot and RT-PCR assay results have demonstrated that PI3Kδ isoforms were highly expressed in endocrine/solid tumor patient specimens and cell lines.

**Results:**

Differential *PIK3CD-S/PIK3CD-L* expression profiles were identified in a panel of endocrine/solid tumor cells. SiRNA knockdown of *PIK3CD-L* or *PIK3CD-S* differentially inhibits AKT/mTOR signaling in PCa, breast, colon and lung cancer cell lines. Moreover, siRNA knockdown of *PTEN* increased PI3Kδ levels and activated AKT/mTOR signaling, while overexpression of *PTEN* reduced PI3Kδ levels and inhibited AKT/mTOR signaling in cancer cells. Intriguingly, PI3Kδ-S levels remained unchanged upon either siRNA knockdown or overexpression of PTEN. Taken together, these results suggested that PTEN negatively regulates PI3Kδ-L and its downstream AKT/mTOR signaling, while PI3Kδ-S promotes AKT/mTOR signaling without regulation by PTEN. Lastly, PI3Kδ inhibitor Idelalisib and SRPK1/2 inhibitor SRPIN340 were employed to assess their efficacies on inhibiting the PI3Kδ-expressing endocrine/solid tumors. Our results have shown that Idelalisib effectively inhibited PI3Kδ-L (but not PI3Kδ-S) mediated AKT/mTOR signaling. In contrast, SRPIN340 reversed the aberrant mRNA splicing, thereby inhibiting AKT/mTOR signaling. *In-vitro* functional assays have further demonstrated that a combination of Idelalisib and SRPIN340 achieved a synergistic drug effect (with drastically reduced cell viabilities/growths of tumor spheroids) in inhibiting the advanced tumor cells.

**Conclusion:**

In summary, our study has suggested a promising potential of utilizing PI3Kδ-S (an oncogenic isoform conferring drug resistance and exempt from PTEN regulation) as a prognostic biomarker and drug target in advanced endocrine cancers.

## Introduction

Phosphatidylinositol-3-kinase (PI3K)/AKT signaling regulates important cellular events, including cell proliferation, survival, mobility, metabolism and angiogenesis (1, 2). Hyperactivation of PI3K/AKT signaling is involved in the development of cancers and treatment resistance (3). Therefore, targeting the modulators/components of this pathway has attracted attention as promising therapeutic options (4, 5). PI3K is a lipid and protein kinase that converts phosphatidylinositol 4,5-bisphosphate (PIP2) to phosphatidylinositol 3,4,5-trisphosphate (PIP3) (6). Class I PI3Ks are heterodimers that consist of a catalytic subunit (PI3Kα, PI3Kβ, or PI3Kδ) and a regulatory subunit (p85α, p85β, or p55γ). PI3Kα and PI3Kβ are found to be ubiquitously expressed, while PI3Kδ is frequently enriched in leukocytes. PTEN is a lipid phosphatase that dephosphorylates/antagonizes the PI3K product, PIP3 (7, 8). PTEN negatively regulates PI3K and inhibits PI3K/AKT signaling, consequently suppressing cell growth, proliferation and migration in cancers (9–12). Conversely, loss of PTEN causes an accumulation of PIP3 and leads to a constitutive activation of PI3K/AKT pathway, leading to cell transformation and development of various cancers (6, 12–14).

Overexpression of PI3Kδ is frequently involved in hematologic malignancies such as chronic lymphocytic leukemia (CLL), multiple myeloma (MM), lymphoma, non-Hodgkin’s lymphoma (NHL), acute myeloid leukemia (AML), and acute promyelocytic leukemia (APL) (15). Emerging evidence has suggested that PI3Kδ is also overexpressed in a wide array of solid tumors, including PCa, breast cancer, ovarian cancer, colon cancer, lung cancer, liver cancer, glioblastoma, and neuroblastoma (16–18). Specifically, PI3Kδ is overexpressed in colorectal cancer (CRC), and ectopic expression of *PIK3CD* (gene encoding PI3Kδ) significantly activates the AKT signaling and promotes CRC cell growth, migration and invasion *in vitro* and tumor growth *in vivo* (19). Upregulation of PI3Kδ was found in hepatocellular carcinoma (HCC), and the higher expression levels of PI3Kδ were correlated with early relapse (20) and poorer survival rates (21) in HCC patients. PI3Kδ has also been shown to regulate the biochemical and bioinformatic properties of breast cancers. For instance, upregulation of PI3Kδ promotes cell migration, invasion, and spheroid growth of breast cancer cell lines (22, 23). Overexpression of PI3Kδ was also detected in primary neuroblastomas and cell lines. In contrast, siRNA knockdown of *PIK3CD* resulted in reduced cell proliferation and enhanced cell apoptosis in neuroblastoma cells (24). In addition, PI3Kδ is highly expressed in various glioblastomas. SiRNA knockdown of *PIK3CD* resulted in a significant reduction of cell invasion and migration in glioblastoma cell lines (25). Taken together, these studies have suggested PI3Kδ as a potential biomarker and drug target for solid tumors.

Alternative splicing is a post-transcriptional process to generate alternative mRNA transcripts that encodes structurally and sometimes also functionally distinct protein isoforms. Genomic analysis such as deep sequencing studies have further revealed that >95% of human genes undergo alternative splicing at the pre-mRNA level (26–28). Emerging evidence has revealed the critical function roles of alternative splicing in cancer development and progression (29–31). Previously, our genomic data have revealed >2,500 alternative splicing events between African American (AA) vs. European American (EA) prostate cancer (PCa) specimens. Several oncogenes and tumor suppressor genes (i.e. *PIK3CD, FGFR3, TSC2, ITGA4, MET, NF1, BAK1*, and *RASGRP2*) were identified with differential splicing patterns in AA vs. EA PCa (32). Notably, *PIK3CD-S* splice variant exhibits more oncogenic properties than the full-length *PIK3CD-L*. Also, PI3Kδ-S (encoded from *PIK3CD-*S) demonstrated a higher kinase activity, more proliferative/invasive capacities, enhanced activation of AKT/mTOR signaling, when compared to the full-length PI3Kδ-L (32, 33). Additionally, molecular modeling, ATP-competitive assays and cell-free kinase assays have further suggested/confirmed that PI3Kδ-S (lacking critical drug binding sites in its catalytic domain due to exon 20 skipping) is more resistant to the PI3K/PI3Kδ inhibitors, when compared to PI3Kδ-L (33).

In this study, we aimed to evaluate the potential of utilizing the aberrant PI3Kδ-S (or *PIK3CD-S* splice variant) as a precision biomarker and to further develop PI3Kδ-S as a novel drug target for PCa and other endocrine/solid tumors. Specifically, immunohistochemistry (IHC), immunofluorescence, RT-PCR and western blot assays were performed to examine the expression profiles of PI3Kδ-S/PI3Kδ-L (or *PIK3CD-S/PIK3CD-L*) in a panel of patient samples and cell lines derived from endocrine/solid tumors, including PCa, breast, pancreatic, colon and lung cancers. Second, IHC and western blot analyses were used to investigate the expression correlation and regulatory roles between PTEN and PI3Kδ or PI3Kδ-S. Third, siRNA knockdown of *PIK3CD-L* and/or *PIK3CD-S* followed by western blot assays were conducted to investigate the inhibitory effects on AKT/mTOR signaling in endocrine/solid tumor cells. Lastly, Idelalisib and SRPK1/2 inhibitor SRPIN340 were used as single agents or in combination to evaluate the therapeutic effects of direct targeting of PI3Kδ and/or modulating splicing mechanisms on inhibiting endocrine/solid tumors expressing *PIK3CD-L/PIK3CD-S*. In summary, this study provided a systematic analysis of PI3Kδ-L and PI3Kδ-S expression profiles in different endocrine/solid tumor specimens and cell lines. By applying approches of molecular/cellular biology, biochemistry, histology and *in-vitro* functional assays, our current study implicated a potential of using PI3Kδ-S as a prognostic biomarker and/or therapeutic target for endocrine cancers.

## Methods

### Cell lines and culture conditions

PCa **(**22Rv1, PC-3, LNCaP, MDA PCa 2b, DU-145 and C4-2B), breast (MDA MB 231 and MCF-7), colon (HT-29 and SW620), and lung (A549 and H1299) cancer cell lines were used in this study. All the cancer cell lines were authenticated and purchased from ATCC (Manassas, VA, USA). 22Rv1, LNCaP, H1299 and MCF-7 were cultured in RPMI-1640 with 10% fetal bovine serum (FBS), PC-3 and A549 were cultured in DMEM with 10% FBS, MDA PCa 2b were cultured in BRFF-HPC1 with 20% FBS, DU-145 was cultured in EMEM with 10% FBS, C4-2B was cultured in Advanced DMEM/F12 with 10% FBS, HT-29 was cultured in McCoy’s with 10% FBS, SW620 and MDA MB 231 were cultured in L-15 with 10% FBS. Cells were maintained at 37°C in a 5% CO_2_ incubator.

### Tissue microarrays (TMA)

To perform immunohistochemistry (IHC), different TMAs were used. First, TMAs containing PCa samples and adjacent normal prostate tissues were used to evaluate the PI3Kδ, PI3Kδ-S, PTEN and AMACR expression levels. The TMAs were purchased from US Biomax Inc. (catalog# PR1921b, Derwood, MD, USA). The TMAs contained total of 192 cores, with 80 cases of adenocarcinoma, 8 adjacent normal prostate tissues from PCa and 8 prostate tissues from normal individuals (duplicate cores of each case were printed on this PCa TMA). The pathological features of the cancerous cores were ranging from Gleason Scores of 2 + 3 to 5 + 5. To evaluate the expression levels of PI3Kδ, and PI3Kδ-S in various endocrine and solid tumors, TMAs containing tumor samples derived from patients diagnosed with PCa, breast cancer, lung cancer, colon cancer, and pancreas cancer specimens (catalog# BC000119b, US Biomax, Derwood, MD, USA) were used. The TMA contained 38 patient specimens from each of breast cancer, lung squamous cell carcinoma, colon adenocarcinoma, prostate adenocarcinoma and pancreas adenocarcinoma (single core per case). The cores were ranging from grades 1 to 3.

### Immunohistochemistry (IHC) assays

The protocol for IHC assay was adapted/modified from our previous studies (34, 35). Briefly, TMA slides were first deparaffinized in xylene, followed by immersion in xylene/alcohol (1:1) solution and rehydrated through graded alcohols (100%, 95%, 70% and 50% of alcohol, respectively) to distilled water. Antigen retrieval was performed using EnVision FLEX target retrieval solution from Agilent technologies (Carpinteria, CA, USA). Thereafter, peroxidase block was added dropwise and incubated for 30 min at room temperature. The slides were then washed with 1×PBS twice, blocked with 2.5% BSA/1×PBS for 30 min at room temperature. After discarding blocking buffer, TMAs were incubated with the primary antibody (1:100–1:200 dilutions in 2.5% BSA/1×PBS) at 4°C overnight. The TMAs were then washed with 1×PBS twice, incubated with HRP-conjugated secondary antibody (Dako, Carpinteria, CA, USA) for 30 min, and the HRP was detected by diaminobenzidine (DAB; Dako, Carpinteria, CA, USA). TMAs were counterstained with Mayer’s hematoxylin (Sigma, St. Louis, MO, USA), and mounted with glycergel mounting medium (Dako, Carpinteria, CA, USA). IHC images were captured using Pannormic Midi Digital Scanner (3DHISTECH Ltd., Budapest, Hungary) and visualized using CaseViewer program (3DHISTECH, Budapest, Hungary). The analysis and quantification of IHC images were performed using ImageJ software (NIH, Bethesda, MD, USA), as described in our previous study (34). The statistical analysis was performed using ANOVA with Tukey’s *post-hoc* test for the multiple comparisons. The PTEN, PI3Kδ, PI3Kδ-S and AMACR antibodies were purchased from Cell Signaling Technology (Waltham, MA, USA), Invitrogen (Waltham, MA, USA) and Agilent Technologies (Santa Clara, CA, USA), respectively.

### Immunofluorescence assays

First, the 2D monolayer cancer culture was established in a glass bottom dish (Cellvis, CA, USA) with an initial density of 5,000 cells, and the 3D spheroid culture was established in a Nunclon Sphera-treated 96-well plate (catalog# 174925, Thermo Fisher Scientific, Waltham, MA, USA) with an initial density of 500-2,000 cells/well according to manufacturer’s protocol. After growing for 5 days, the cells were washed with 1×PBS and then fixed in 4% paraformaldehyde for 15 min at room temperature. The fixed cells were then permeabilized with 0.1% Triton X-100 for 10 min, and blocked with 2% BSA/1×PBS for 1 h at room temperature. Primary antibodies against PI3Kδ and PI3Kδ-S were added, and the cultures were further incubated overnight at 4°C. Thereafter, the cells were washed three times with 1×PBS, and followed by incubating with Alexa-Fluor-488-conjugated anti-mouse and Alexa-Fluor-594-conjugated anti-rabbit antibodies (Invitrogen, Waltham, MA, USA). After 1h, the cells were washed three times with 1×PBS and mounted with Prolong glass antifade NucBlue Stain from Invitrogen (cat# P36981, Waltham, MA, USA). The fluorescence signals were visualized using fluorescence microscope (Olympus, MA, USA) or Stellaris confocal microscope (Leica, Deerfield, IL, USA). The fluorescence images of 2D cultures were captured by CellSens V1.18 software (Olympus, Waltham, MA, USA) and analyzed by using ImageJ (NIH, Bethesda, MD, USA). For 3D spheroid cultures, the fluorescence images were captured and processed using Leica Application Suite X (LAS X) software (Leica, Deerfield, IL, USA). The intensities of fluorescence signals were quantified using ImageJ software (NIH, Bethesda, MD, USA). Specifically, the fluorescence images (green or red fluorescence images) were converted to 16-bit images and the fluorescence signals were adjusted/captured using threshold for measuring their integrated densities (IntDen). The intensity of PI3Kδ (green) were defined as 100% based on the IntDen of PI3Kδ signals in each experimental group, and the intensity of PI3Kδ-S in each group was determined by normalization of PI3Kδ-S to PI3Kδ intensities, using equation of (IntDen of PI3Kδ-S)/(IntDen of PI3Kδ) × 100%.

### RT-PCR assay

The wild-type PCa (22Rv1, PC-3, LNCaP, DU-145, C4-2B, MDA PCa 2b), breast cancer (MDA MB 231 and MCF-7), colon cancer (HT-29 and SW620), and lung cancer (A549 and H1299) cell lines were seeded at a density of 1×10^5^ cells/well in 6 well plates. The cancer cells were cultured at 37°C in a 5% CO_2_ incubator. After culturing the cells for 48 h, the cells were subjected for RNA purification. Selective endocrine/solid tumor cell lines (22Rv1, LNCaP, MDA PCa 2b, HT29, A549 and MCF-7) were grown and then treated with vehicle, 25 μM of Idelalisib, 25 μM of SRPIN340, or combination of Idelalisib (25 μM) and SRPIN340 (25 μM) for 48 h at 37°C. Thereafter, the cells were harvested and subjected to RNA purification. RNA purification was performed using miRNeasy kit from Qiagen (Germantown, MD, USA) according to the manufacturer’s protocol. The purified RNA samples were reversely transcribed to cDNA using iScript reverse transcription supermix (Bio-Rad, Hercules, CA). The reverse transcription reactions were performed as follows: 25°C for 5min, 46°C for 50min, then 95°C for 1min. The resulting cDNA samples were used for PCR reactions to examine *PIK3CD-L* and *PIK3CD-S* expression profiles, and *EIF1AX* was used as an endogenous control. The primers used for the PCR reactions were listed in **Supplementary Table S1**. The *S/L* ratio of each cell line was determined by calculating the signal density of *PIK3CD-S*/density of *PIK3CD-L* from the RT-PCR assays. The RT-PCR results of *EIF1AX* were used as endogenous controls for *PIK3CD-L* and *PIK3CD-S* expression levels in different PCa cell lines. Primers PIK3CD-f, PIK3CD-r1, EIF1AX-f and EIF1AX-r were used in the PCR reactions.

### Western blot assay

1×10^6^ cells of 22Rv1, LNCaP, MDA PCa 2b, HT-29, A549 and MCF-7 cell lines were seeded in 10-cm plates and the cancer cells were incubated at 37°C in a 5% CO_2_ incubator. After incubation for 24 h, the cells were under different treatments of siRNAs, plasmid or drugs and incubated for additional 48 h. For siRNA knockdown or gene overexpression experiments, the cancer cells were transfected with nonsense/scrambled (NS) siRNA, 1 μM of si*PIK3CD*, 1 μM of si*PIK3CD-L* (targeting exon 20 of *PIK3CD*), 1 μM of si*PIK3CD-S* (targeting to the junction region between exon 19 and 21 of *PIK3CD-S*), si*PTEN*, or pcDNA3-FLAG PTEN. For the drug treatment experiments, the cancer cells were treated with vehicle, 25 μM of Idelalisib, 25 μM of SRPIN340, or a combination of Idelalisib (25 μM) and SRPIN340 (25 μM). The drug concentrations of Idelalisib and SRPIN340 were determined based on our previous study (33). After the treatments, the cancer cells were harvested and the protein lysates were extracted using M-PER extraction reagent with protease and phosphatase inhibitor cocktail (Thermo Fisher Scientific, Waltham, MA, USA) according to manufacturer’s protocol. Equal amounts of proteins were used based on the quantification using a BCA assay kit (Thermo Fisher Scientific, Waltham, MA, USA), and the proteins were separated by electrophoresis using NuPAGE 4-12% Bis-Tris gels (Invitrogen, Waltham, MA, USA). The gels were transferred to PVDF membranes (Bio-Rad, Hercules, CA, USA) then the PVDF membranes were incubated with primary antibodies and secondary antibodies. The membranes were then incubated with SuperSignal ECL substrates (Thermo Fisher Scientific, Waltham, MA, USA) and the signals were detected using ChemiDoc XRS system (Bio-Rad, Hercules, CA, USA). The primary and secondary antibodies used in the study were mouse monoclonal antibody against PI3Kδ (Santa Cruz Biotechnology, TX, USA), polyclonal antibody against PI3Kδ-S (Invitrogen, Waltham, MA, USA), monoclonal rabbit antibodies against PTEN, pAKT, AKT, pS6, S6, β-actin, and anti-rabbit/mouse IgG-HRP antibodies (Cell Signaling Technology, Waltham, MA, USA).

### BrdU-labeling cell proliferation assay

22Rv1, LNCaP, PC-3, MDA PCa 2b, HT-29, A549 and MCF-7 cells were seeded at density 5,000 cells/well in 96-well culture plates. The cells were incubated overnight and then were either transfected with NS, *siPIK3CD*, *siPIK3CD-L* or *siPIK3CD-S*. The cells were incubated for another 24 h, then were subjected to bromodeoxyuridine (BrdU) incorporation assay to analyze cell proliferation capacities. The assays were performed using BrdU Cell Proliferation Assay Kit (Sigma-Aldrich, St. Louis, MO, USA) as described by manufacturer’s protocol and our previous studies (32, 36). The measurements were based on the absorbances at dual wavelengths of 450 nm and 540 nm using Multiskan FC microplate photometer (Thermo Scientific, Waltham, MA, USA).

### MTT assays of the spheroid cultures under drug treatments

The endocrine/solid tumor cell lines 22Rv1, LNCaP, MDA PCa 2b, HT-29, A549 and MCF-7 were seeded at densities of 500-2,000 cells/well in the 96-well Nunclon Sphera-treated plates (catalog# 174925, Thermo Fisher Scientific, Waltham, MA, USA) containing DMEM/10% FBS media. The tumor spheroids were first incubated at 37°C in a 5% CO_2_ incubator for 2 days, then 25 μM of Idelalisib, 25 μM of SRPIN340, or a combination of Idelalisib (25 μM) and SRPIN340 (25 μM) were added as treatments for additional 5 days. For monitoring the spheroid growths, each well was imaged every day using Olympus IX73 microscope (Olympus, Bartlett, TN, USA) and then the spheroid diameter, area and circularity were measured by ImageJ. For measuring the cell viabilities of spheroids under different drug treatments, the CellTiter 96 Non-Radioactive Cell Proliferation Assay reagent (Promega, Madison, WI, USA) was added to each well and incubated with the spheroids for 3 h at 37°C, then the solubilization solution was added. After incubation for 1h, the samples were detected by the Multiskan FC microplate photometer (Thermos Fisher Scientific, Waltham, MA, USA) at the wavelength of 570 nm. The data were analyzed by GraphPad Prism 9 program (GraphPad, La Jolla, CA, USA).

## Results

### PI3Kδ-L and PI3Kδ-S were highly expressed in PCa specimens and cell lines

To evaluate whether PI3Kδ and/or PI3Kδ-S splice variant can serve as a potential biomarker, a series of patient samples and cell lines derived from PCa, breast cancer, colorectal, lung and/or pancreatic cancers were subjected to IHC, western blot, and RT-PCR assays for examining the expression profiles of PI3Kδ/PI3Kδ-S and *PIK3CD-L/PIK3CD-S* at protein and mRNA levels, respectively.

**Figure 1:**
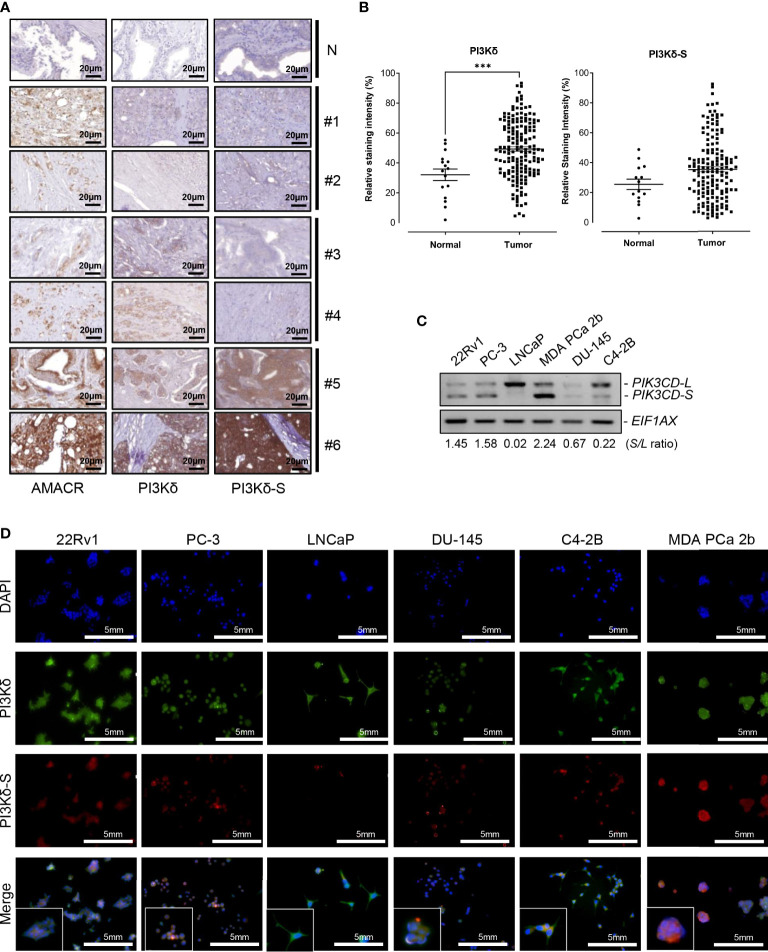
Expression levels of PI3Kδ and PI3Kδ-S splice isoforms, at protein and RNA levels, in a panel of PCa patient samples and cell lines. **(A)** IHC staining assays showing the expression levels of AMACR, PI3Kδ and PI3Kδ-S in six PCa specimens (#1 to #6) on the TMAs. N: normal prostate tissues. Scale bar: 20 μm. **(B)** Quantification of PI3Kδ and PI3Kδ-S intensities from the IHC images of the PCa patients and normal controls on TMAs. Significantly different (***p-value < 0.001) PI3Kδ intensities between PCa specimens and normal prostate tissues. The *p*-values were determined based on the two-tailed student t-test. Data values represent mean ± SEM. **(C)** RT-PCR assays to examine the expression profiles of full-length *PIK3CD-L* and *PIK3CD-S* splice variant in PCa cell lines 22Rv1, PC-3, LNCaP, MDA PCa 2b, DU-145, and C4-2B. The *S/L* ratios were determined as described in Methods. **(D)** Immunofluorescence assays results showing the expression of representative PI3Kδ (green fluorescence) and PI3Kδ-S (red fluorescence) signals in PCa (22Rv1, PC-3, LNCaP, DU-145, C42B, and MDA PCa 2b) cells. Nuclei were counterstained with DAPI (blue), and the Merged images were obtained by overlaying PI3Kδ, PI3Kδ-S with DAPI signals. Scale bar: 5 mm.

First, we conducted IHC assays on a TMA containing 160 cancerous and 16 adjacent normal tissues from 80 PCa patients, and 16 normal prostate tissues from 8 healthy individuals. The Gleason scores (GS) of the PCa samples on the TMA were ranging from 2 + 3 to 5 + 5. To assess the expression levels of PI3Kδ isoforms in the PCa samples on the TMA, three independent IHC assays were performed to examine the expression profiles of α-methylacyl CoA racemase (AMACR, a potential PCa biomarker), PI3Kδ (using a general PI3Kδ antibody recognizing all PI3Kδ isoforms) and PI3Kδ-S splice isoform (using PI3Kδ-S specific antibody). As shown in **Figure 1A**, three groups of PCa samples were revealed. Group 1 (patient #1 and #2 as examples) represented the PCa expressing AMACR, but not PI3Kδ nor PI3Kδ-S. Group 2 (patient#3 and #4 as examples) represented the PCa expressing AMACR and PI3Kδ, but not PI3Kδ-S. Group 3 (patient#5 and #6 as examples) represented the PCa expressing AMACR, PI3Kδ, and PI3Kδ-S. Specifically, the IHC assay results revealed that almost 100% of PCa samples on the TMA expressed low to high levels of AMACR. Among all the AMACR-positive PCa samples, 82.5% (132 out of 160) and 60% (97 out of 160) samples expressed PI3Kδ and PI3Kδ-S, respectively. Notably, the PCa specimens displaying medium levels of AMACR also expressed medium levels of PI3Kδ, but not necessarily expressed PI3Kδ-S (i.e. patient #3 and #4, **Figure 1A**). Whereas, all the PCa specimens displaying high levels of AMACR expressed high levels of both PI3Kδ and PI3Kδ-S (i.e. patient #5 and #6, **Figure 1A**). Quantification of IHC images have further confirmed that PI3Kδ and PI3Kδ-S expression levels were much higher in PCa specimens than normal prostate tissues (**Figure 1B**). Next, we further evaluated the correlation between PI3Kδ and PI3Kδ-S expression levels and PCa aggressiveness (i.e. PCa with different Gleason scores). Quantification of IHC staining results demonstrated that comparable total PI3Kδ (PI3Kδ-L + PI3Kδ-S) levels were observed in PCa with different disease grades (from GS 2 + 2 to 5 + 4). However, PI3Kδ-S expression levels appeared to positively correlate with the Gleason scores of the PCa specimens (**Supplementary Figure S2**). Taken together, total PI3Kδ (with comparable levels as AMACR in PCa specimens) and PI3Kδ-S (highly expressed in PCa samples with higher Gleason scores and higher AMACR levels) may potentially serve as precision biomarkers for PCa diagnosis and prognosis, respectively.

A series of PCa cell lines with different pathological features were further subjected to RNA purifications and RT-PCR assays to examine the expression profiles of *PIK3CD-L* (that encodes full-length PI3Kδ) and *PIK3CD-S* splice variant. PC-3 and DU-145 are androgen receptor (AR) negative PCa and represent androgen-independent PCa cell line models, LNCaP is an AR-positive and androgen-dependent PCa derived from lymph node. C4-2B (developed from LNCaP) and 22Rv1 are known as castration resistant prostate cancer (CRPC) cell lines, and MDA PCa 2b is an androgen-independent PCa cell line derived from bone metastasis of an African American (AA) patient. The RT-PCR results have revealed that the androgen-independent PCa cell lines MDA PCa 2b, PC-3, 22Rv1 and DU-145 have higher *PIK3CD-S/PIK3CD-L* (*S/L*) ratios, 2.24, 1.58, 1.45, and 0.67. The androgen-sensitive/dependent LNCaP predominately expressed full-length *PIK3CD-L*, with a lowest *S/L* ratio of 0.02. Compared to LNCaP, C4-2B (a CRPC cell line derived from LNCaP) expressed a higher level of *PIK3CD-S* with a *S/L* ratio of 0.22 (11 time higher than the *S/L* ratio in parental cell line LNCaP) (**Figure 1C**). These results indicated that higher *PIK3CD-S* expression levels may correlate with more aggressive PCa phenotypes. Additionally, western blot analysis was conducted to verify the protein expression levels of PI3Kδ and PI3Kδ-S in PCa cell lines. Similar to RT-PCR results in **Figure 1C**, PI3Kδ-S was expressed in 22Rv1, PC-3, MDA PCa 2b, and C4-2B. Notably, MDA PCa 2b still demonstrated a highest PI3Kδ-S/PI3Kδ-L ratio (S/L ratio of 2.23). In contrast, LNCaP predominately expressed PI3Kδ-L (with S/L ratio of 0.37), and DU-145 expressed lowest level of PI3Kδ-L and PI3Kδ-S isoforms (**Supplementary Figures S1A, B**).

Next, we conducted immunofluorescence staining assays to visualize and verify the expression levels of PI3Kδ and PI3Kδ-S in the PCa cell lines described above. As shown in **Figure 1D**, the six PCa cell lines demonstrated differential PI3Kδ (green fluorescence) and PI3Kδ-S (red fluorescence) expression profiles/levels. Specifically, 100% of 22Rv1 expressed total PI3Kδ (green fluorescence) and PI3Kδ-S (red fluorescence). The ratio of red to green fluorescence intensities was ~3:5 (PI3Kδ-S: PI3Kδ-L+PI3Kδ-S), which is equal to PI3Kδ-S/PI3Kδ-S (S/L) ratio of 1.44. PC-3 expressed red to green fluorescence ratio of ~1:2, which is equal to a S/L ratio of 0.96. LNCaP expressed high level of PI3Kδ but very low level of PI3Kδ-S, with an average red to green ratio of ~1:9, equal to S/L ratio of 0.12. In DU-145 cells, the expression levels of PI3Kδ (green signals) was approximately 2.6-fold higher than the PI3Kδ (red signals). Therefore, the average red to green ratios in DU-145 cells was ~1:2.6 (which is equal to S/L ratio of 0.64). C4-2B cells expressed three-fold higher intensities of total PI3Kδ (green signals) than PI3Kδ-S (red signals), indicating that the red to green ratio was ~1:4.2 that is equal to S/L ratio of 0.32. Whereas, MDA PCa 2b cells expressed high level of red fluorescence (PI3Kδ-S) signals, with ~70% of fluorescence intensities when compared to the green signals (total PI3Kδ). The results suggested that MDA PCa 2b exhibits an average red to green ratio of ~7:10, which is equal to S/L ratio of 2.6 (**Figure 1D**; **Supplementary Table S2A**). Taken together, the immunofluorescence assays showed similar trends of S/L ratios as the results obtained from the RT-PCR assays (**Figure 1C**) in these six PCa cell lines.

### Both PI3Kδ-L and PI3Kδ-S were expressed in endocrine and solid tumor specimens and cell lines

**Figure 2:**
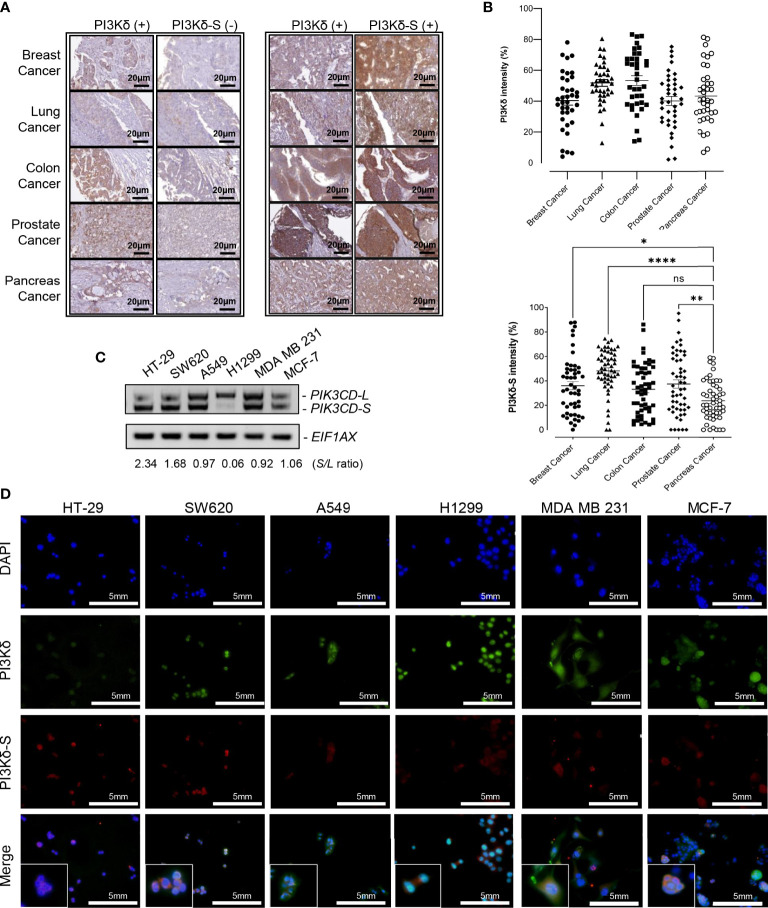
PI3Kδ-L and PI3Kδ-S splice isoform are generally overexpressed in solid tumors, including endocrine cancers. **(A)** Representative IHC staining images for breast, lung, colon, prostate and pancreatic cancers. A subset of PI3Kδ-positive patient samples derived from the endocrine/solid tumors expressed PI3Kδ-S splice isoform (right panel). Scale bar: 20 μm. **(B)** Quantification of PI3Kδ and PI3Kδ-S levels from the IHC staining images of breast, prostate, pancreatic, lung, and colon cancers specimens on the TMAs. The mean and SEM values were shown on the dot plots. **(C)** RT-PCR assays to examine the expression profiles of *PIK3CD-L* and *PIK3CD-S* splice variant in colon (HT-29 and SW620), lung (A549 and H1299), and breast (MDA MB 231 and MCF-7) cancer cell lines. The *S/L* ratios were determined as described in Methods. **(D)** Immunofluorescence assays showing the expression levels of PI3Kδ (green fluorescence) and PI3Kδ-S (red fluorescence) in colon (HT29 and SW620), lung (A549 and H1299), and breast (MDA MB 231 and MCF-7) cancer cells. Nuclei were counterstained with DAPI (blue), and Merge images were generated by overlaying PI3Kδ and PI3Kδ-S with DAPI signals. Scale bar: 5 mm. ns: not significant. *p-value < 0.05, **p-value < 0.01, and ****p-value < 0.0001 were determined based on ANOVA with Dunnett's post hoc test.

To further investigate whether PI3Kδ-L and PI3Kδ-S are generally expressed in solid tumors, IHC assays were performed on TMAs containing cancerous specimens derived from endocrine cancer (breast, prostate, and pancreatic cancer), lung cancer and colorectal cancer patients. First, IHC results have revealed that PI3Kδ was expressed in majority of breast, prostate, pancreatic, lung and colon cancer specimens on the TMA (**Figure 2A**). Similar to the observation in **Figure 1**, PI3K signals were detected in most of the cancer specimens, while PI3Kδ-S levels were detected in subsets of the breast, lung, colon, prostate and pancreatic cancer specimens. Specifically, 70-80% of cancer specimens expressed both PI3Kδ (PI3Kδ-L + PI3Kδ-S) and PI3Kδ-S splice isoform (**Figure 2A**, right panel). Whereas, 20-30% of the PI3Kδ-positive cancer samples did not express PI3Kδ-S (**Figure 2A**, left panel). Quantification of IHC images further revealed that PI3Kδ was expressed at comparable levels (with average intensities ranging from 40% to 50%) in these cancers (**Figure 2B**, top panel). IHC quantification also demonstrated that comparable expression levels of PI3Kδ-S were detected in breast, lung and colon cancers (with average intensities ranging from 35% to 45%), while a statistically lower average PI3Kδ-S level (with average intensity of ~20%) was detected in pancreatic cancer specimens (**Figure 2B**, lower panel).

To further evaluate *PIK3CD-L* and *PIK3CD-S* expression profiles in the *in-vitro* endocrine/solid tumor cell models, the RNA samples purified from breast cancer cell lines (MDA MB 231 and MCF-7), colon cancer cell lines (HT-29 and SW620), and lung cancer cell lines (A549 and H1299) were subjected to RT-PCR assays. The RT-PCR results have shown differential *PIK3CD-L* and *PIK3CD-S* expression profiles between these cancer cell lines. Specifically, HT-29 and SW620 expressed higher levels of *PIK3CD-S* than *PIK3CD-L* (with *S/L* ratios of 2.34 and 1.68), A549, MDA MB 231 and MCF-7 expressed comparable levels of *PIK3CD-L* and *PIK3CD-S* (with *S/L* ratios of 0.97, 0.92 and 1.06, respectively), while H1299 predominately expressed *PIK3CD-L* (with *S/L* ratio of 0.06) (**Figure 2C**).

Next, immunofluorescence assays were performed to visualize/verify the expression levels of PI3Kδ and PI3Kδ-S in the colon, lung and breast cancer cell lines described above. As shown in **Figure 2D**, HT-29, SW620, A549, H1299, MDA MB 231 and MCF-7 expressed differential PI3Kδ (green fluorescence, PI3Kδ-L + PI3Kδ-S) and PI3Kδ-S (red fluorescence) expression profiles. Specifically, HT-29 exhibited a red to green fluorescence ratio of ~1:1.3 (equal to S/L ratio of 3.17), SW620 exhibited a red to green fluorescence ratio of ~0.75:1 (equal to S/L ratio of 2.85), A549 exhibited a red to green fluorescence ratio of ~1:3.5 (equal to S/L ratio of 0.41), H1299 expressed a red to green fluorescence ratio of ~1:8 (equal to S/L ratio of 0.14), MDA MB 231 demonstrated a red to green fluorescence ratio of ~1:3 (equal to S/L ratio of 0.43), and MCF-7 expressed a red to green fluorescence ratio of ~1:2.5 (equal to S/L ratio of 0.7) (**Figure 2D**; **Supplementary Table S2B**). Consistent with the IHC and RT-PCR results, the immunofluorescence assays again confirmed that both PI3Kδ-L and PI3Kδ-S were expressed in HT-29 and SW620, A549, H1299, MDA MB 231 and MCF-7 with differential *S/L* ratios.

### Correlation of PI3Kδ and PI3Kδ-S expression levels with cancer aggressiveness

To evaluate the correlation between PI3Kδ/PI3Kδ-S expression profiles and tumor aggressiveness, we reviewed the IHC staining results of PI3Kδ and PI3Kδ-S in cancers with different pathological features/states. Interestingly, comparable PI3Kδ expression levels (relative staining intensities ranging from 50-57%) were detected in PCa patient with Gleason Scores (GS) of 2 + 3, 3 + 3, 4 + 3, and 5 + 4 (**Supplementary Figure S3A**, top panel). In contrast, elevated PI3Kδ-S level seemed to be correlated with high-grade PCa. Specifically, the expression levels of PI3Kδ-S increased from GS 2 + 3, 3 + 3, 4 + 3 to 5 + 4 (relative staining intensities from 10.8, 14.97, 36.44, to 79.60%, **Supplementary Figure S3A**, bottom panel). Similarly, comparable PI3Kδ expression levels were observed in endocrine/solid tumor patients with different tumor grades (i.e. G1 vs. G2 vs. G3) (i.e. the relative PI3Kδ staining intensities, **Supplementary Figures S3B–E**, top panels). However, expression levels of PI3Kδ-S splice isoform were increased from G1, G2, to G3 in patients diagnosed with breast, pancreatic, colon and lung cancers (i.e. the gradually increased PI3Kδ-S staining intensities in G1, G2, and G3, **Supplementary Figures S3B–E**, bottom panels). Taken together, these results again suggested that PI3Kδ may serve as a potential diagnostic biomarker for endocrine/solid tumors in general, while PI3Kδ-S splice isoform may particularly serve as a prognostic biomarker for predicting cancer aggressiveness.

### SiRNA knockdown of PIK3CD-L and/or PIK3CD-S inhibits ATK/mTOR signaling in endocrine/solid tumors

**Figure 3:**
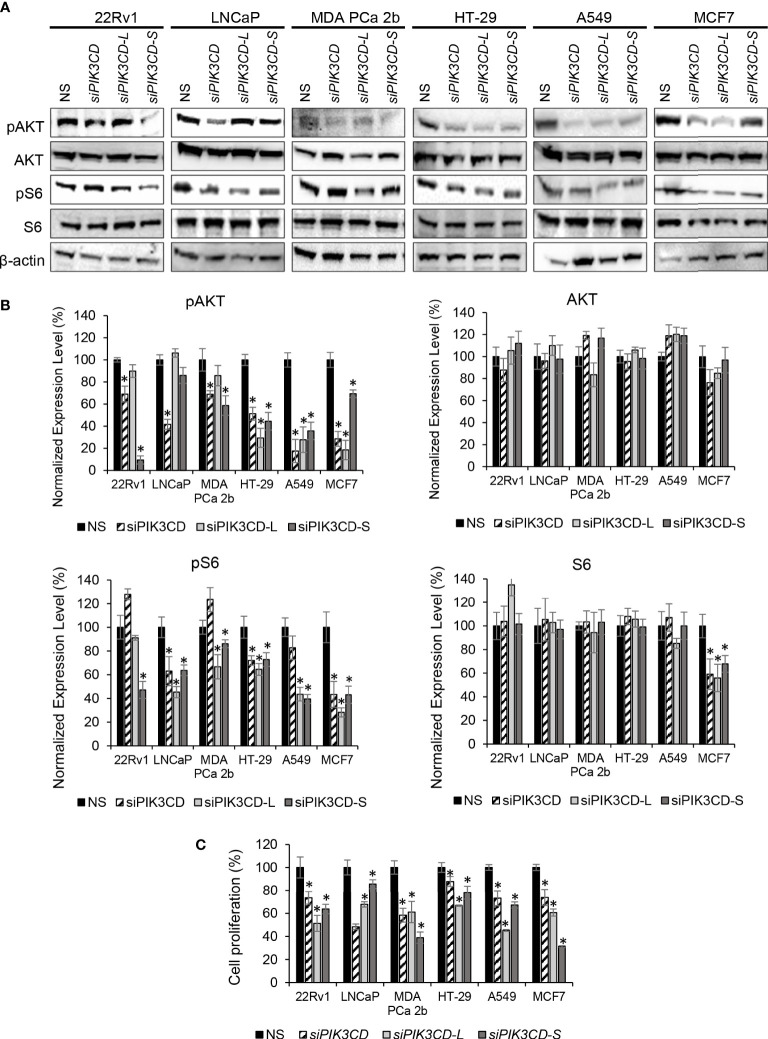
Inhibitory effects of AKT/mTOR signaling upon siRNA knockdown of total *PIK3CD* (*PIK3CD-L* and its splice variants)*, PIK3CD-L* or *PIK3CD-S* in PCa, colon, lung and breast cancer cell lines. **(A)** Western blot analysis of pAKT, AKT, pS6 and S6 in the cancer cells transfected with nonsense siRNA (NS), *siPIK3CD, siPIK3CD-L* or *siPIK3CD-S*. β-actin was used as an endogenous control for the western blot analysis of pAKT, AKT, pS6 and S6. **(B)** Quantification of pAKT, AKT, pS6 and S6 levels (of all experimental groups in A, normalized to NS controls) from 3-4 independent western blot results. **(C)** BrdU-labeling cell proliferation assays in PCa cells transfected with NS, *siPIK3CD*, *siPIK3CD-L* or *siPIK3CD-S*. All the bar graphs in **(B, C)** were presented as mean ± SD, and the significances (*p-value < 0.05, in siRNA knockdown vs. NS) were calculated based on one-way ANOVA with Dunnett’s *post hoc* tests.

Previously, we reported that expression of *PIK3CD-L* or *PIK3CD-S* splice variant promotes oncogenic activation of AKT/mTOR signaling (32, 33). To verify the functional effects of *PIK3CD-L* and *PIK3CD-S* in endocrine and solid tumors expressing *PIK3CD-L* and *PIK3CD-S* splice variant, the cancer cell lines were transfected with nonsense siRNA (NS), *siPIK3CD*, *siPIK3CD-L* (siRNA targeting exon 20 of *PIK3CD*), or *siPIK3CD-S* (siRNA targeting junction of exon 19 and 21) for 48 h then the transfected cells were harvested and subjected to western blot analysis for examining the protein levels of the AKT/mTOR signaling components. Androgen-sensitive PCa (LNCaP, with lowest *PIK3CD-S/PIK3CD-L* ratio), androgen-independent PCa (22Rv1 and MDA PCa 2b, with high *PIK3CD-S/PIK3CD-L* ratios), colon, lung and breast cancer cell lines (HT-29, A549 and MCF-7, with higher *PIK3CD-S/PIK3CD-L* ratios among each cancer types) were selected as *in-vitro* endocrine/solid tumor cell models for this experiment. First, RT-PCR assays confirmed the efficiencies and specificities of siRNA knockdown using *siPIK3CD*, *siPIK3CD-L* and *siPIK3CD-S*. Second, the cell viability assays performed upon siRNA knockdowns further confirmed that no toxicities were observed upon siRNA treatments (**Supplementary Figure S4**). Upon the efficient/specific knockdown of total *PIK3CD, PIK3CD-L* or *PIK3CD-S*, the phosphorylation states of AKT are significantly reduced upon siRNA knockdown of total *PIK3CD, PIK3CD-L* or *PIK3CD-S* vs. NS, in all the endocrine/solid tumor cell lines. Additionally, a statistically significant reduction in the phosphorylation states of S6 were observed in *siPIK3CD*, *siPIK3CD-L* and *siPIK3CD-S* vs. NS transfected cancer cell lines (**Figures 3A, B**). Followed by siRNA knockdown of NS, total *PIK3CD*, *PIK3CD-L* or *PIK3CD-S*, all the cancer cells were harvested and then subjected to BrdU-based cell proliferation assays. As shown in **Figure 3C**, the cell proliferative capacities were significantly reduced upon knockdown of *PIK3CD*, *PIK3CD-L* or *PIK3CD-S*. These results suggested that: 1) both oncogenic *PIK3CD-L* and *PIK3CD-S* variants promote the activation of AKT/mTOR signaling pathway; and 2) molecular targeting of *PIK3CD-L* and/or *PIK3CD-S* inhibits ATK/mTOR signaling and subsequently suppresses cell proliferation, potentially serving as a novel therapeutic strategy for treating aggressive endocrine/solid tumors.

### Correlation between PTEN and PI3Kδ expression levels in PCa specimens

**Figure 4:**
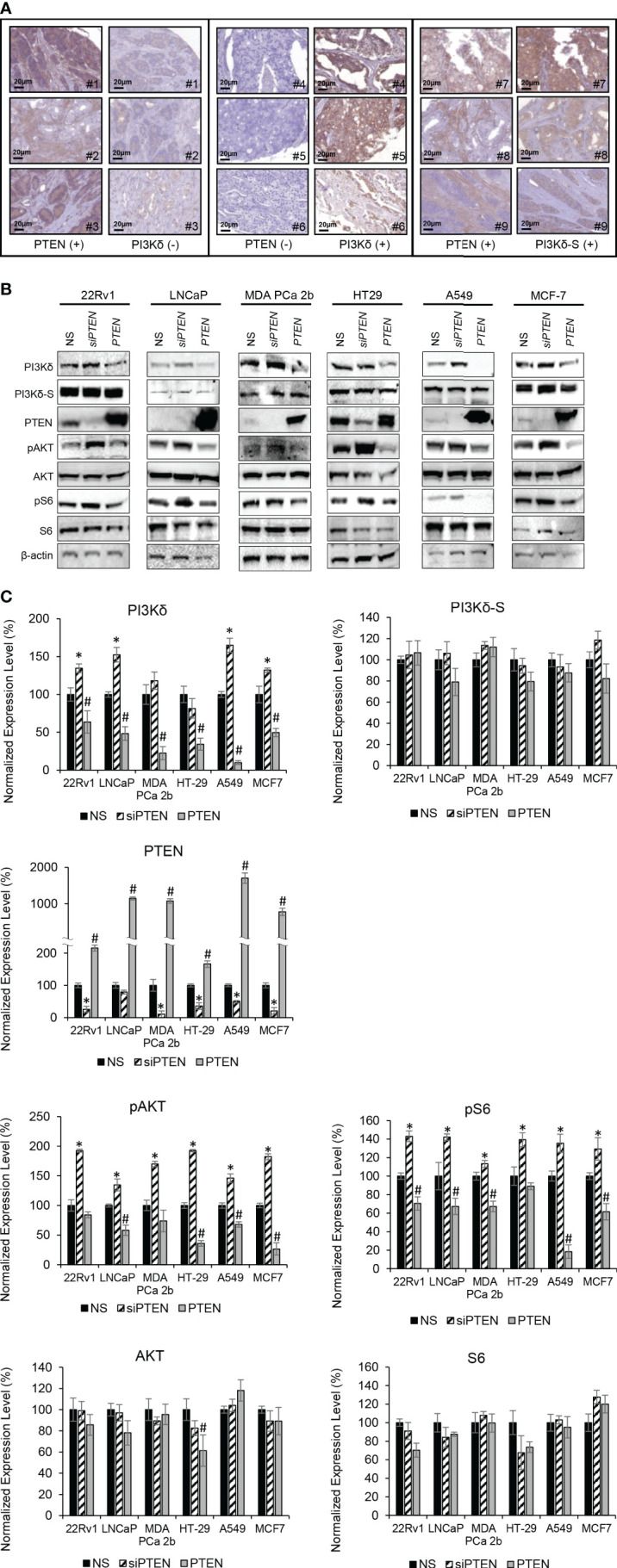
PTEN negatively regulates expression levels of total PI3Kδ, but not PI3Kδ-S splice isoform. **(A)** Representative IHC staining images showing three categories of PTEN/PI3Kδ expression profiles/patterns in PCa patient specimens. Specifically, the PCa patients expressing high levels of PTEN but low levels of PI3Kδ (left panel). The PCa patients expressing low levels of PTEN but high levels of PI3Kδ (middle panel). The PCa patients expressing high levels of PTEN as well as high levels of PI3Kδ-S. **(B)** Western blot analysis of PI3Kδ, PI3Kδ-S, PTEN, pAKT, AKT, pS6, S6 and β-actin levels in 22Rv1, LNCaP, MDA PCa 2b, HT29, A549 and MCF-7 cells transfected with nonsense/scrambled (NS) siRNA, *siPTEN* or pcDNA3-FLAG-PTEN plasmid (indicated as *PTEN*). **(C)** Quantification of PI3Kδ, PI3Kδ-S, PTEN, pAKT, AKT, pS6 and S6 levels (of all experimental groups in **(B)**, normalized to NS controls) from 3-4 independent western blot results. The bar graphs were presented as mean ± SD, and the significances (*p-value < 0.05 in *siPTEN* vs. NS, and ^#^ *p*-value < 0.05 in *PTEN* vs. NS) were calculated based on one-way ANOVA with Tukey’s *post hoc* tests.

*PTEN* is a primary tumor suppressor gene, and it is frequently inhibited, deleted or loss-of-function in PCa. Previous studies have shown that overexpression of *PTEN* causes a decrease in *PIK3CA* expression, while knockdown of *PTEN* increases the *PIK3CA* expression (37, 38). To verify whether there is a negative correlation/regulation between PTEN and PI3Kδ or PI3Kδ-S, IHC analysis of PTEN, PI3Kδ and PI3Kδ-S protein expression were performed using TMAs containing same cohort of the PCa specimens. Among the 160 PCa specimens, 111 PCa samples exhibited negative correlations between PTEN and PI3Kδ protein levels. Specifically, 98 PCa specimens expressed very low to no PTEN, but expressed high levels of PI3Kδ. In contrast, 13 PCa specimens expressed PTEN but not PI3Kδ. The representative IHC images of PTEN and PI3Kδ with negative correlations were shown in **Figure 4A** (left and middle panels). Surprisingly, IHC results have further revealed that 30 PCa specimens simultaneously expressed PTEN and PI3Kδ-S splice isoform (representative images in **Figure 4A**, right panel), implicating that PI3Kδ-S expression may be not regulated/suppressed by PTEN in these PCa patients.

### Modulation of PTEN expression negatively regulates expression levels and activities of full-length PI3Kδ, but not PI3Kδ-S splice isoform

Except PC-3, LNCaP and C4-2B (*PTEN*-negative PCa cell lines), other endocrine/solid tumor cells expressed low to high levels of PTEN (**Supplemental Figure S1**). It appears that PTEN expression levels (**Supplementary Figure S1**) were negatively correlated with the *PIK3CD-L* (but not *PIK3CD-S*) expression levels (**Figures 1C**, **2C**). For example, *PIK3CD-L* was highly expressed in the PTEN-negative PCa cell lines (PC-3, LNCaP and H1299), while *PIK3CD-L* was underexpressed/inhibited in the PTEN-overexpressing DU-145 and HT-29. In contrast, *PIK3CD-S* was expressed in all the PTEN-positive PCa cell lines (**Figures 1C**, **2C**; **Supplementary Figure S1**). To further assess the functional roles of PTEN in regulating PI3Kδ and PI3Kδ-S expression, western blot analyses were performed in selected/representative cancer cells transfected with NS, *siPTEN*, or pcDNA3-FLAG-PTEN plasmid (indicated as *PTEN*). The western blot results showed that siRNA knockdown of *PTEN* significantly increased the protein levels of PI3Kδ, while overexpression of *PTEN* drastically reduced PI3Kδ expression levels in 22Rv1, LNCaP, MDA PCa 2b, HT29, A549, and MCF-7 cells (**Figures 4B, C**). Moreover, phosphorylation states of AKT and S6 were significantly enhanced upon siRNA knockdown of *PTEN* in cancer cells, suggesting AKT/mTOR signaling is activated upon loss of *PTEN*. In contrast, *PTEN* overexpression significantly inhibits phosphorylation of AKT and S6 in all the six cancer cell lines (**Figures 4B, C**). These results confirmed that PTEN negatively regulates PI3Kδ expression and suppresses AKT/mTOR signaling. Interestingly, PI3Kδ-S expression levels remained comparable/unchanged between NS-transfected, *siPTEN*-transfected and PTEN-overexpressing cells (**Figures 4B, C**). These results again suggested that PI3Kδ-S protein expression is independent from regulation/suppression by PTEN.

### SRPIN340 induces RNA splice switching and inhibits AKT/mTOR signaling in combination with Idelalisib in endocrine/solid tumor

Idelalisib is an ATP-competitive inhibitor that specifically targets PI3Kδ and has potent anticancer effects against PI3Kδ-expressing cancer cells (39). Our previous studies have demonstrated that overexpression of *PIK3CD-S* splice variant in PCa confers AA PCa resistance to PI3Kδ inhibitor, such as Idelalisib (32, 33). We also uncovered that the synthesis of aberrant *PIK3CD-S* splice variant is likely mediated by the splicing factor SRSF2, and inhibition of SRSF2 by SRPK1/2 inhibitor SRPIN340 significantly sensitizes AA PCa to Idelalisib (33).

**Figure 5:**
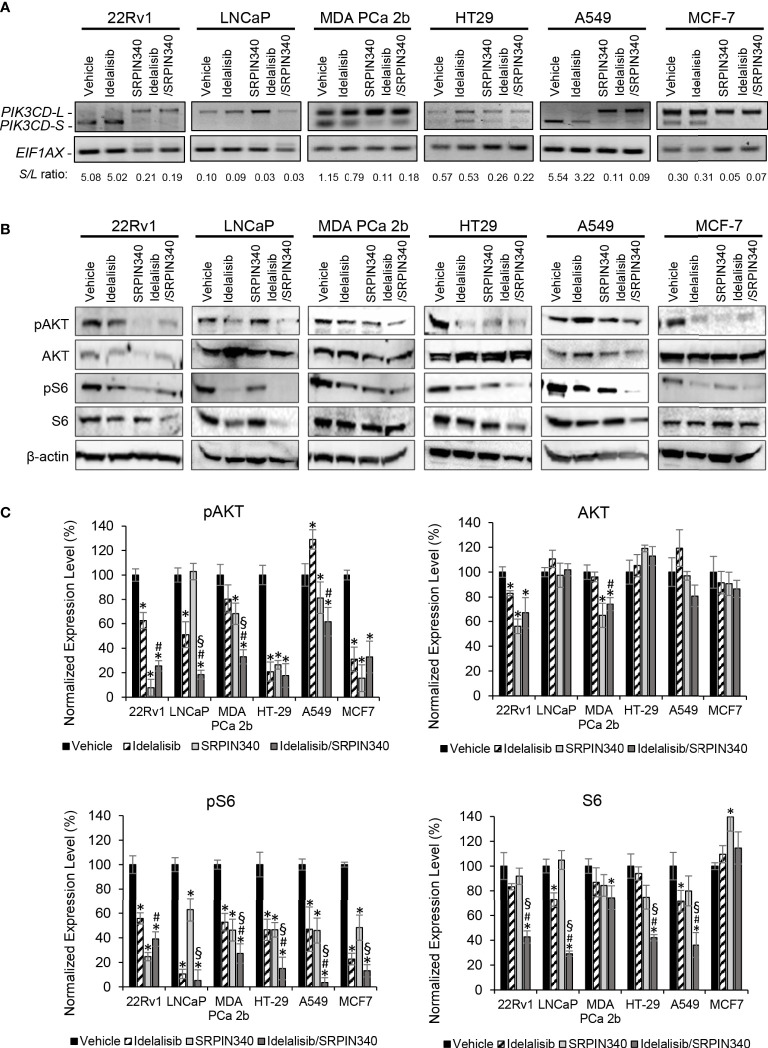
SRPK1/2 inhibitor SRPIN340 reverses the aberrant splicing and sensitizes endocrine/solid tumor cells to PI3Kδ inhibitor Idelalisib. **(A)** RT-PCR assays for examining the *PIK3CD-L* and *PIK3CD-S* expression profiles in 22Rv1, LNCaP, MDA PCa 2b, HT29, A549 and MCF-7 cells in the presence of vehicle, 25 µM of Idelalisib, 25 µM of SRPIN340 and a combination of 25 µM Idelalisib and 25 µM SRPIN340. *EIF1AX* was used as an endogenous control for the RT-PCR assays. The *S/L* ratios were determined as described in Methods. **(B)** Western blot analysis of pAKT, AKT, pS6 and S6 protein levels in 22Rv1, LNCaP, MDA PCa 2b, HT29, A549 and MCF-7 cells. The western blot images of pAKT, AKT, pS6 and S6 were representative blot images from 3-4 independent repeats. The β-actin was used as an endogenous protein control. **(C)** Quantification of pAKT, AKT, pS6 and S6 levels (of all experimental groups in **(B)**, normalized to NS controls) from 3-4 independent western blot results. The bar graphs were presented as mean ± SD, and the significances (*p-value < 0.05 in drug treatment vs. vehicle, ^#^ *p-*value < 0.05 in Idelalisib/SRPIN340 vs. Idelalisib, and ^§^ *p-*value < 0.05 in Idelalisib/SRPIN340 vs. SRPIN340) were calculated based on one-way ANOVA with Tukey’s *post hoc* tests.

Except LNCaP, all the endocrine/solid tumor cell lines (22Rv1, MDA PCa 2b, HT29, A549, and MCF-7) expressed moderate to high levels of PI3Kδ-S (**Figures 1C**, **2C**). In theory, SRPIN340 treatment could inhibit the synthesis of *PIK3CD-S* variant through blocking the exon 20 skipping in *PIK3CD* pre-mRNA, thereby enriching the *PIK3CD-L* (which is sensitive to Idelalisib treatment) in these cancer cells. To validate this hypothesis, these cancer cell lines were grown and treated with vehicle, Idelalisib, SRPIN340, or a combination of Idelalisib and SRPIN340. After treatment for 48 hr, the cancer cells were harvested and subjected to RNA purification and RT-PCR assays. As anticipated, an ‘RNA splice switching’ pattern was observed in SRPIN340 vs vehicle treatments among the endocrine/solid tumor cell lines that express *PIK3CD-S* (22Rv1, MDA PCa 2b, HT-29, A549, and MCF-7). Compared to the vehicle control, SRPIN340 treatment inhibits the synthesis of *PIK3CD-S*, evident from the RT-PCR results (gel images in **Figure 5A**) and the *S/L* ratios were drastically decreased in all the cell lines (significant reduction of *S/L* ratios in SRPIN340 vs. vehicle, in **Figure 5A**). In contrast, no changes in *S/L* profiles/ratios were found in Idelalisib vs. vehicle treatments. These results strongly suggest that SRPIN340 inhibits *PIK3CD-S* synthesis and causes an ‘RNA splice switching’ to convert *PIK3CD-S* to *PIK3CD-L*, producing the full-length PI3Kδ that is sensitive to Idelalisib.

Next, we examined the inhibitory effects of AKT/mTOR signaling pathway in the presence of vehicle, Idelalisib, SRPIN340, or Idelalisib/SRPIN340 combination in the same cancer cell lines. As shown in **Figures 5B, C**, pAKT and pS6 were significantly decreased in LNCaP (that predominately expressed PI3Kδ-L) in response to Idelalisib or Idelalisib/SRPIN340 combination compared to vehicle control. In contrast, no inhibition of pAKT and pS6 was observed in LNCaP cells under SRPIN340 treatment (reflecting the fact that negligible *PIK3CD-S* was present in LNCaP). For other endocrine/solid tumors (22Rv1, MDA PCa 2b, HT-29, A549, and MCF-7) that expressed both PI3Kδ-L and PI3Kδ-S, the phosphorylation states of AKT and S6 were significantly reduced in response to either Idelalisib or SRPIN340 treatment. And notably, the phosphorylation of AKT and pS6 were almost completely inhibited in cells treated with the Idelalisib/SRPIN340 combination (**Figures 5B, C**).

### Combination of Idelalisib and SRPIN340 effectively inhibits cancer spheroids and exhibits potent cytotoxicity in PIK3CD-S expressing cancers

**Figure 6:**
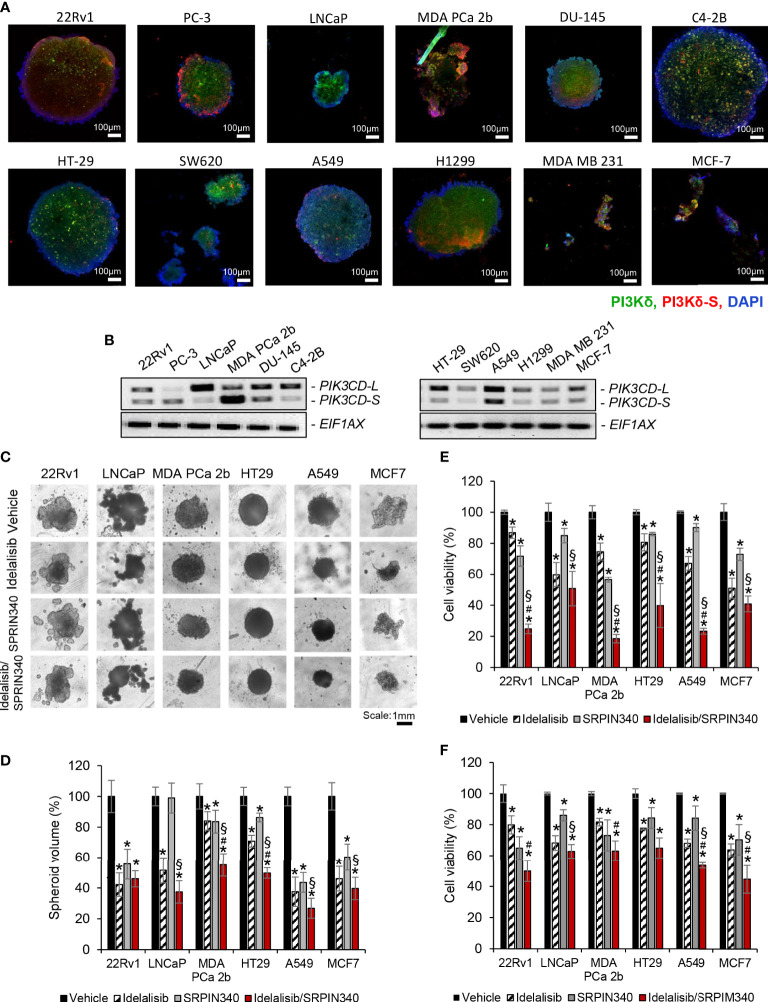
Cell viability assays of and the endocrine/solid tumor cells under treatments of PI3Kδ inhibitor and/or SRPK1/2 inhibitor. Immunofluorescence assays were employed to visualize the expression levels of total PI3Kδ (green fluorescence) and PI3Kδ-S splice isoform (red fluorescence) in the tumor spheroids developed from **(A)** PCa cell lines (22rv1, PC3, LNCaP, MDA PCa 2b DU-145 and C4-2B), and endocrine/solid tumor cell lines (HT29, SW620, A549, H1299, MDA MB 231 and MCF-7). Nuclei were counterstained with DAPI (blue). **(B)** RT-PCR results revealing the expression profiles of *PIK3CD-L* and *PIK3CD-S* in all the tumor spheroids. **(C)** The bright-field images of tumor spheroids under treatment of vehicle, Idelalisib, SRPIN340, or Idelalisib/SRPIN340 combination for 5 days. **(D)** The average volumes of the endocrine/solid tumor spheroids after treatment of vehicle, Idelalisib, SRPIN340, or Idelalisib/SRPIN340 combination for 5 days. The volumes of spheroids were calculated based on the equation: V = 4/3πR^3^, where V is volume and R is the radius averaged from 3-4 spheroids. The volume of the vehicle-treated spheroid in each cell line was defined as 100%. Therefore, the relative spheroid volume under treatment was determined by normalizing to its control (i.e. volume of drug-treated spheroid/volume of vehicle-treated spheroid × 100%). Significances (**p-value < 0.05 in drug treatment vs. vehicle, ^#^ *p-*value < 0.05 in Idelalisib/SRPIN340 vs. Idelalisib, and ^§^ *p-*value < 0.05 in Idelalisib/SRPIN340 vs. SRPIN340) were calculated based on one-way ANOVA with Tukey’s *post hoc* tests. Data values represent mean ± SD from 3-4 independent experiments. MTT assays for **(E)** 2D monolayer cultures and **(F)** 3D spheroid cultures of 22Rv1, LNCaP, MDA PCa 2b, HT-29, A549 and MCF-7 cells in the presence of vehicle, Idelalisib (25 µM), SRPIN340 (25 µM) and combination therapy (25 µM Idelalisib and 25 µM SRPIN340). Significances (*p-value < 0.05 in drug treatment vs. vehicle, ^#^ *p-*value < 0.05 in Idelalisib/SRPIN340 vs. Idelalisib, and ^§^ *p-*value < 0.05 in Idelalisib/SRPIN340 vs. SRPIN340) were determined based on one-way ANOVA with Tukey’s *post-hoc* tests. Data values represent mean ± SEM from 5-6 independent experiments.

To evaluate the drug efficacies in the context of tumor microenvironment, 3D spheroid cultures in presence of vehicle, Idelalisib, SRPIN340, or Idelalisib/SRPIN340 combination were established. First, the 3D spheroid cultures developed from endocrine/solid tumor cell lines (22Rv1, PC-3, LNCaP, MDA PCa 2b, DU-145, C4-2B, HT-29, SW620, A549, H1299, MSA MB 231, and MCF-7) were assessed on day 5 using immunofluorescence assays to visualize the expression levels of PI3Kδ and PI3Kδ-S splice isoform. The green fluorescence represented the total PI3Kδ signals (total of PI3Kδ-L and PI3Kδ-S) and red fluorescence reflected the PI3Kδ-S signals (**Figure 6A**; **Supplementary Figure S5**). The immunofluorescence results from the 3D spheroids have demonstrated similar expression levels/patterns of PI3Kδ-L and PI3Kδ-S as we observed in regular 2D cell cultures (**Figures 1D**, **2D**).

Second, all the spheroid cultures were harvested for RNA purifications and RT-PCR assays, to examine their expression profiles of *PIK3CD-L* and *PIK3CD-S*. The expression profiles of *PIK3CD-L* and *PIK3CD-S* in 22Rv1, PC-3, LNCaP, MDA PCa 2b, DU-145, C4-2B, A549, H1299, MDA MB 231, and MCF-7 spheroid cultures were similar to the expression patterns from the corresponding 2D cultures (**Figure 6B**). However, HT-29 and SW620 spheroids expressed much lower *PIK3CD-S* levels than their 2D monolayer cultures (**Figure 6B** vs. **Figure 2C**).

Next, we examined the efficacies of PI3Kδ inhibitor and SRPK1/2 inhibitor (as single agents or in combination) on inhibiting the 2D monolayer and 3D spheroids *in vitro*. Specifically, the tumor spheroids were cultured in the presence of vehicle, 25 μM of Idelalisib, 25 μM of SRPIN340, or a combination of Idelalisib and SRPIN340. After drug treatments for 5 days, the tumor spheroids exhibited differential responses upon different treatments. In general, all the tumor spheroids responded to Idelalisib with significant reduction (i.e. 30-60% reduction) in tumor spheroid sizes/volumes, except MDA PCa 2b (**Figures 6C, D**). After SRPIN340 treatment for 5 days, all the tumor spheroids exhibited decreased spheroid sizes/volumes (i.e. 20-60% reduction), except LNCaP (**Figures 6C, D**). The differential drug responses reflect the fact that LNCaP almost exclusively expressed PI3Kδ-L (not responding to SRPIN340) while MDA PCa 2b predominantly expressed PI3Kδ-S (that is resistant to Idelalisib). However, significant reduction in tumor spheroid volumes of all cancer lines were observed in the presence of Idelalisib/SRPIN340 combination, suggesting a synergistic drug effect of combining Idelalisib with SRPIN340 on inhibiting the PI3Kδ-L/-S expressing tumor spheroids (**Figures 6C, D**).

MTT assays were further performed to examine the drug effects on the cell viabilities of the regular 2D cultures and 3D spheroid cultures. Specifically, 22Rv1, LNCaP, MDA PCa 2b, HT-29, A549, and MCF-7 cells were treated with vehicle, Idelalisib, SRPIN340, or Idelalisib/SRPIN340 combination for 48 h (for 2D cultures) or 5 days (for 3D spheroid cultures) then subjected to MTT assays. As shown in **Figure 6E**, the cell viabilities were significantly reduced in response to 25 μM of Idelalisib or 25 μM of SRPIN340 in all cell lines, except LNCaP in the presence of SRPIN340. Notably, a significant synergistic drug effect (with 50-80% decreases in cell viabilities) was observed in all cancer cell lines when treated with Idelalisib/SRPIN340 combination (**Figure 6E**). Similar to the MTT assay results in the 2D cell cultures, Idelalisib or SRPIN340 has exerted moderate inhibitory capacities (with 15-35% decrease in cell viabilities) in all spheroid cultures. However, synergistic drug effects (with 40-55% reduction in cell viabilities) were observed in all the tumor spheroids, except HT-29 spheroid (that predominately expressed *PIK3CD-L*), treated with Idelalisib/SRPIN340 combination (**Figure 6F**).

## Discussion

Emerging evidence has revealed that PI3Kδ is expressed not only in hematologic cancers, but also highly expressed in solid tumors (16–18). Particularly, previous studies have shown that PI3Kδ is overexpressed in PCa cell lines (DU-145, 22Rv1, PC-3 (18), LNCaP and MDA PCa 2b (33)), breast cancer lines (MDA MB 231 and MCF-7 (22)), colon cancer lines (SW620 and SW480 (40)) and lung cancer cell lines (A549, H1975, PC9 and H1650 (41)). Consistent with the previous studies, our data again confirmed that PI3Kδ is expressed in LNCaP, DU-145, PC-3, 22Rv1, MDA PCa 2b, MDA MB 231, MCF-7, SW620 and A549. Additionally, C4-2B (a castration resistant prostate cancer cell line) and H1299 (lung cancer cell line) have also shown high levels of PI3Kδ. Notably all these endocrine/solid tumor cell lines also expressed PI3Kδ-S splice isoform (based on our IHC, immunofluorescence, and RT-qPCR analyses). To date, this was the first attempt to investigate the expression profiles of full-length PI3Kδ-L and PI3Kδ-S splice isoform in endocrine/solid tumor patient samples and cell lines. Given the fact that PI3Kδ and PI3Kδ-S exhibit differential oncogenic activities, our data suggested that a potential of utilizing the *PIK3CD-S/PIK3CD-L* (or PI3Kδ-S/PI3Kδ-L) expression profile as an index to evaluate the tumor aggressiveness in endocrine cancers.

To further evaluate the potential of PI3Kδ-L and/or PI3Kδ-S as a diagnostic/prognostic biomarker, a survival analysis was performed. By employing the PanCanSurvPlot program (https://smuonco.shinyapps.io/PanCanSurvPlot/), possible correlations between PI3Kδ expression levels and cancer patient survival rates have been revealed (**Supplementary Method**). Specifically, higher expression levels of PI3Kδ (based on the GEO database and RNA-seq data from TCGA database) appears to be correlated with poorer survival rates in selected patient cohorts with endocrine cancers (prostate, breast, pancreatic, ovarian, endometrial and cervical) and solid tumors (colon and lung cancer) (**Supplementary Figure S6**). Our previous studies demonstrated that *PIK3CD-S* is a more oncogenic splice variant (compared to *PIK3CD-*L), and expression of PI3Kδ-S confers a drug resistance phenotype in PCa (32, 33). Further bioinformatic efforts (i.e. retrieving RNAseq data from TCGA database to precisely define *PIK3CD-L* and *PIK3CD-S* expression levels in cancer patients from selected cohorts) may facilitate our understanding on whether *PIK3CD-S/PIK3CD-L* or PI3Kδ-S/PI3Kδ-L expression ratios would correlate with the poorer survival rates and/or disease aggressiveness (i.e. drug resistance, recurrence, metastasis, and etc.) in endocrine cancers.

The tumor suppressor PTEN plays a critical role in regulating PI3K/AKT/mTOR signaling. Mutations and/or loss-off-function in *PTEN* are frequently found in various cancers, including endocrine/solid tumors, such as PCa, breast, colon, and lung cancers (42). A negative regulation between PTEN and PI3K has been highlighted in several cancers. In PCa, it has been shown that PTEN suppresses the expression of *ARID4B*, repressing the transcriptional activation of *PIK3CA* and subsequently inhibiting the PI3K/AKT signaling (37). In human nasopharyngeal carcinoma cells, siRNA knockdown of *PTEN* resulted in upregulation of PI3K (at mRNA and protein levels) and activation of PI3K/AKT signaling, while suppressing tumor suppressor FOXO3a (38). On the other hand, previous study further showed that PI3K levels may also modulate the activities of PTEN. Specifically, siRNA knockdown of *PIK3CD* activated PTEN activity. Whereas, ectopic expression of PI3Kδ resulted in suppression of PTEN activity, consequently suppressing AKT signaling and inhibiting cell proliferation in PCa and breast cancer cells (18, 43). Similar to the previous findings, our results have also confirmed that inhibition of *PTEN* by siRNA caused upregulation of PI3Kδ expression and activation of AKT/mTOR signaling, evident from the increased pAKT and pS6 levels. Conversely, ectopic expression of *PTEN* resulted in suppression of AKT/mTOR signaling (i.e. reduced phosphorylation states of AKT and S6). Additionally, our data have further revealed that PTEN negatively regulates PI3Kδ protein expression level. However, PI3Kδ-S protein expression seems to be independent from regulation of PTEN (**Figure 4**). To date, the mechanisms underlying the PTEN-independent PI3Kδ-S expression remain unknown. One of the possible mechanisms is: PI3Kδ-S expression levels are determined by the synthesis of *PIK3CD-S*, an aberrant splice variant resulted from a SRSF2-mediated exon 20 skipping event in *PIK3CD* pre-mRNA. This aberrant RNA splicing process is independent from regulation by PTEN, and therefore, the PI3Kδ-S levels could solely depend on the activities of SRSF2 in each cell lines. Further investigation of the upstream regulators of PTEN (i.e. p53, EGFR1, PPAR-γ, SPRY2, and etc. (44)) in each cell lines may help to elucidate the molecular mechanisms underlying the PTEN-independent PI3Kδ-S protein expression.

Furthermore, siRNA knockdowns of total *PIK3CD* variants, *PIK3CD-L* and *PIK3CD-S* showed differential inhibitory effects on AKT/mTOR signaling, possibly due to the differential S/L ratios in different cancer cell lines. Overall, *siPIK3CD* knockdown demonstrated good inhibition in AKT/mTOR signaling in general (**Figure 3**). Notably, siRNA knockdown of *PIK3CD-S* has shown superior inhibition of AKT/mTOR signaling (i.e. significant reduction of pAKT and pS6 levels) in 22Rv1 and MDA PCa 2b, reflecting the higher sensitivities upon *siPIK3CD-S* due to their higher *S/L* ratios (1.45 and 2.24, respectively).

Accumulating evidence has suggested that aberrant mRNA splicing may represent one of the genetic mechanisms mediating drug resistance in cancers (29, 31). In this study, we proposed a novel therapy by combining PI3Kδ inhibitor with splicing inhibitor. As shown in **Figures 6C–F**, this drug combination generated a significantly synergistic effect on inhibiting the tumor growths and viabilities in the 2D monolayer and 3D spheroid cultures derived from the endocrine/solid tumor cell lines. To date, this is also the first report to systematically apply SRPK1/2 inhibitor (SRPIN340) for sensitizing the drug resistant endocrine/solid tumors. This drug combination may represent a novel therapy for treating Idelalisib-resistant cancers, with non-hematologic or hematologic origin. Future efforts of employing oligonucleotide therapy (such as antisense oligonucleotides, ASO and splice switching oligonucleotides, SSO), targeting critical splicing modulator (such as SF3B1, a core component in spliceosome), or screening/developing compounds specifically inhibiting PI3Kδ-S activity, may further warrant the development of novel therapies for overcoming the Idelalisib resistance in the PI3Kδ-expressing endocrine cancers.

## Conclusion

In conclusion, our study has revealed that PI3Kδ is overexpressed in endocrine cancer or solid tumors in general. PI3Kδ-S splice isoform exhibits a more oncogenic activity (compared to PI3Kδ-L), and is expressed in subgroups of all the cancers we examined, including PCa, breast, pancreatic, colon and lung cancers. Compared to the full-length PI3Kδ-L, the splice isoform PI3Kδ-S seems to be exempt from the inhibition by PTEN. SRPIN340, a SRPK1/2 inhibitor, reverses the aberrant splicing and sensitizes the advanced endocrine/solid tumors to the PI3Kδ-specific inhibitor, such as Idelalisib. To date, this is the first systematic analysis on the expression profiles of PI3Kδ splice isoforms across different endocrine/solid tumors. The synergistic inhibitory effects of Idelalisib/SRPIN340 combination may pave a new path for developing novel therapeutics for the Idelalisib-resistant endocrine/solid tumors (and hematologic cancers, in theory) that express PI3Kδ-S.

## Data availability statement

The original contributions presented in the study are included in the article/**Supplementary Material**. Further inquiries can be directed to the corresponding author.

## Ethics statement

Ethical approval was not required for the studies on humans in accordance with the local legislation and institutional requirements because only commercially available established cell lines were used. Ethical approval was not required for the studies on animals in accordance with the local legislation and institutional requirements because only commercially available established cell lines were used.

## Author contributions

Conceptualization: B-DW; Methodology: SH, HG, and B-DW; Conducting of experiments and data acquisition: SH, HG, and B-DW; Data analysis and interpretation: SH, HG, and B-DW; Writing—original draft: SH, HG, and B-DW; Writing review and editing: SH and B-DW; Funding acquisition: B-DW; Resources: B-DW; Supervision: B-DW. All authors contributed to the article and approved the submitted version.
