# Supplementary material for: Aberrant PI3Kδ splice isoform as a potential biomarker and novel therapeutic target for endocrine cancers
Source: Front Endocrinol (Lausanne). 2023 Aug 21;14:1190479. doi: 10.3389/fendo.2023.1190479 (PMC10475954; doi:10.3389/fendo.2023.1190479)
Supplement: Supplementary file 1 [file DataSheet_1.pdf]

Supplementary Method

Survival curves for cancer patients expressing high and low levels of PI3Kδ. The survival curves showing the changes of survival rates along the time in cancer patients expressing high and low levels of PI3Kδ were plotted using PanCanSurvPlot program (https://smuonco.shinyapps.io/PanCanSurvPlot/). PanCanSurvPlot retrieves microarray or RNA-sequencing data of cancer patients from the GEO and TCGA databases, and further performs the survival analysis [1]. The program collected a total of 215 cancer-related databases from the GEO and TCGA databases, covering ~45,000 samples from 51 different cancer types and 13 survival outcome datasets. The survival data analyses were performed using Kaplan-Meier method, and long-rank test and univariant Cox proportional hazard regression model was utilized to assess the correlation between gene expression profiles and clinical outcomes. The end users can define the patient groups with high and low expression levels of specific gene, based on the median or optimal cutoff values. In **Supplementary Figure S6**, we selected patient cohorts from PCa, breast, pancreatic, ovarian, endometrial, cervical, colon and lung cancers, and divided cancer patient groups with high and low PI3Kδ expression levels based on 'optimal' cutoff values. The survival curves showing significantly lower survival rates (*p*-values < 0.05, long-rank test and univariant Cox proportional hazard regression) in patients expressing high PI3Kδ vs. low PI3Kδ were plotted.

1. Lin, A., Yang, H., Shi, Y., Cheng, Q., Liu, Z., Zhang, J., & Luo, P. (2022). PanCanSurvPlot: A Large-scale Pan-cancer Survival Analysis Web Application. BioRxiv, 2022.12.25.521884. https://doi.org/10.1101/2022.12.25.521884.

Supplementary Table S1. Primer sequences used for the RT-PCR assays.

Primer ID	Nucleotide sequences
PIK3CD-f	5'-CTGAGCTCTCAGAAGACC-3'
PIK3CD-r1	5'-GCTCGCGGTTGATTCCAA-3'
PIK3CD-r2	5'-AATAGCCAGCACAGGAGAGG-3'
EIF1AX-f	5'-GTACTGGAGAGGGGAGAGCA-3'
EIF1AX-r	5'-TGAAGCTGAGACAAGCAGGA-3'

Supplementary Table S2. Quantification of PI3Kδ-L and PI3Kδ-S expression levels in PCa and other endocrine/solid tumor cell lines. % of green (PI3Kδ) and red (PI3Kδ-S) fluorescence-positive cells were counted by measuring (numbers of green signals/numbers of blue signals × 100%) and (numbers of red signals/numbers of blue signals × 100%) signals from the immunofluorescence assay results in **Figure 1D** and **2D**. Green (PI3Kδ) and red (PI3Kδ-S) fluorescence intensities of each images from immunofluorescence assays in **Figure 1D** and **2D** were quantified using ImageJ, as described in Methods. In each cell line, green (PI3Kδ) fluorescence intensity was defined as 100%. Red (PI3Kδ-S) intensity was quantified and normalized to the green (PI3Kδ) fluorescence intensity in each cell line. The PI3Kδ-L intensities were calculated using equation of: 100% - (% of PI3Kδ-S).

A.

	**22Rv1**	**PC-3**	**LNCaP**	**DU-145**	**C42B**	**MDA PCa 2b**
**PI3Kδ (+) cells, %**	100	100	100	100	100	100
**PI3Kδ-S (+) cells, %**	100	100	33	100	100	100
**PI3Kδ intensity, %**	100	100	100	100	100	100
**PI3Kδ-S intensity, %**	59	49	11	39	24	72
**PI3Kδ-L intensity, %**	41	51	89	61	76	28
**PI3Kδ-S: PI3Kδ**	3:5	1:2	1:9	1:2.5	1:4.2	7.2:10
**PI3Kδ-S/PI3Kδ-L ratio (S/L ratio)**	1.44	0.96	0.12	0.64	0.32	2.6

B.

	**HT-29**	**SW620**	**A549**	**H1299**	**MDA MB 231**	**MCF-7**
**PI3Kδ (+) cells, %**	100	100	100	100	100	100
**PI3Kδ-S (+) cells, %**	100	100	100	100	100	50
**PI3Kδ intensity, %**	100	100	100	100	100	100
**PI3Kδ-S intensity, %**	76	74	29	12	30	41
**PI3Kδ-L intensity, %**	24	26	71	88	70	59
**PI3Kδ-S: PI3Kδ**	1:1.3	0.75:1	1:3.5	1:10	1:3	1:2.5
**PI3Kδ-S/PI3Kδ-L ratio (S/L ratio)**	3.17	2.85	0.41	0.14	0.43	0.70
