# Supplementary material for: Aberrant PI3Kδ splice isoform as a potential biomarker and novel therapeutic target for endocrine cancers
Source: Front Endocrinol (Lausanne). 2023 Aug 21;14:1190479. doi: 10.3389/fendo.2023.1190479 (PMC10475954; doi:10.3389/fendo.2023.1190479)
Supplement: Supplementary file 2 [file DataSheet_2.pdf]

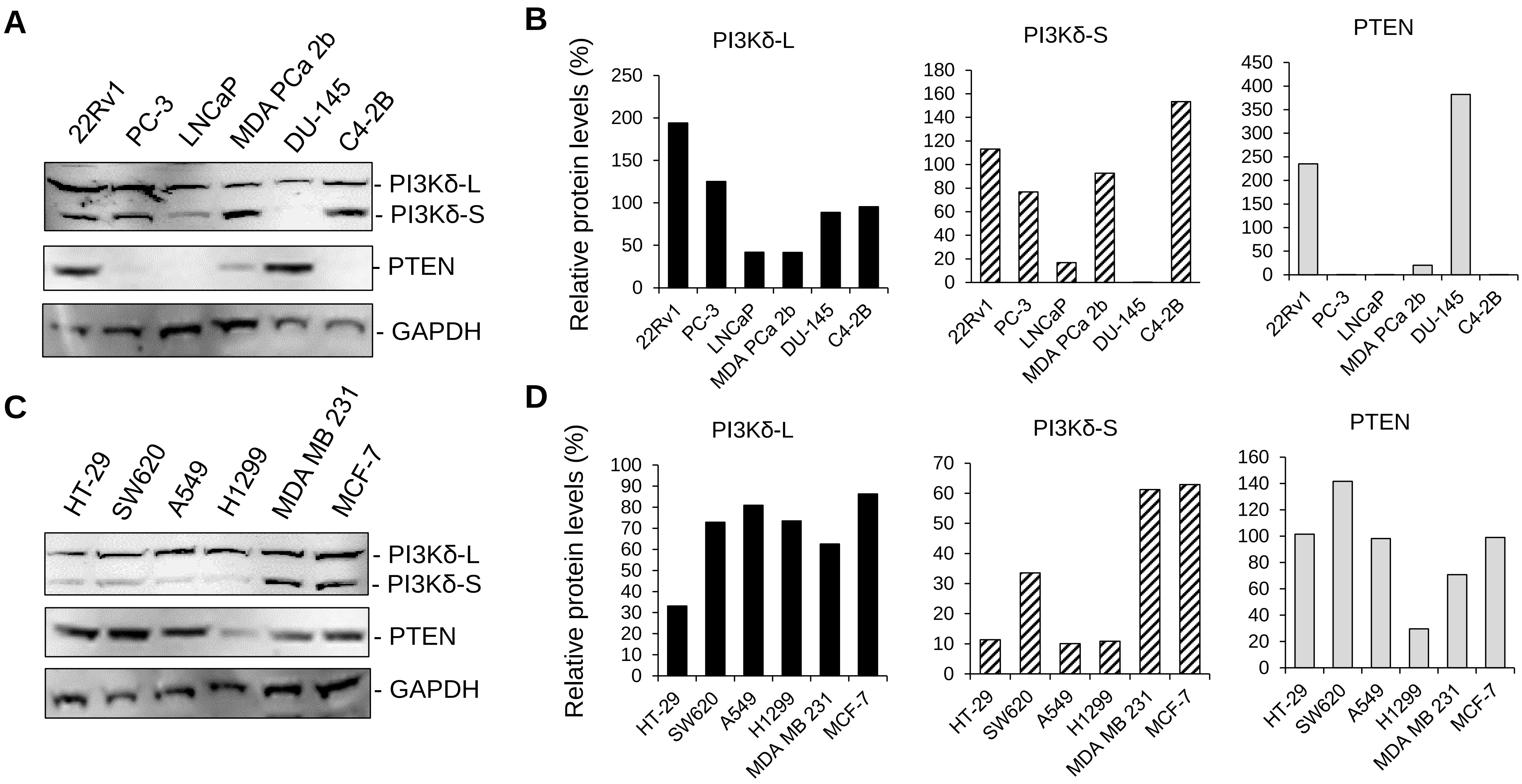

Supplementary Figure S1. Protein levels of PI3Kδ-L, PI3Kδ-S and PTEN in endocrine and solid tumors. (A) Western blots and **(B)** Quantification of PI3Kδ-L, PI3Kδ-S and PTEN expression levels in PCa cell lines 22Rv1, PC-3, LNCaP, MDA PCa 2b, DU-145 and C4-2B. **(C)** Western blots and **(D)** Quantification of PI3Kδ-L, PI3Kδ-S and PTEN expression levels in colon (HT-29, SW620), lung (A549, H1299), and breast (MDA MB 231, MCF-7) cancer cell lines. GAPDH was used as an endogenous control for the western blots (representative images from n=3). The quantification data (in **B** and **D**) were relative protein levels after normalization to GAPDH levels in corresponding cell lines.

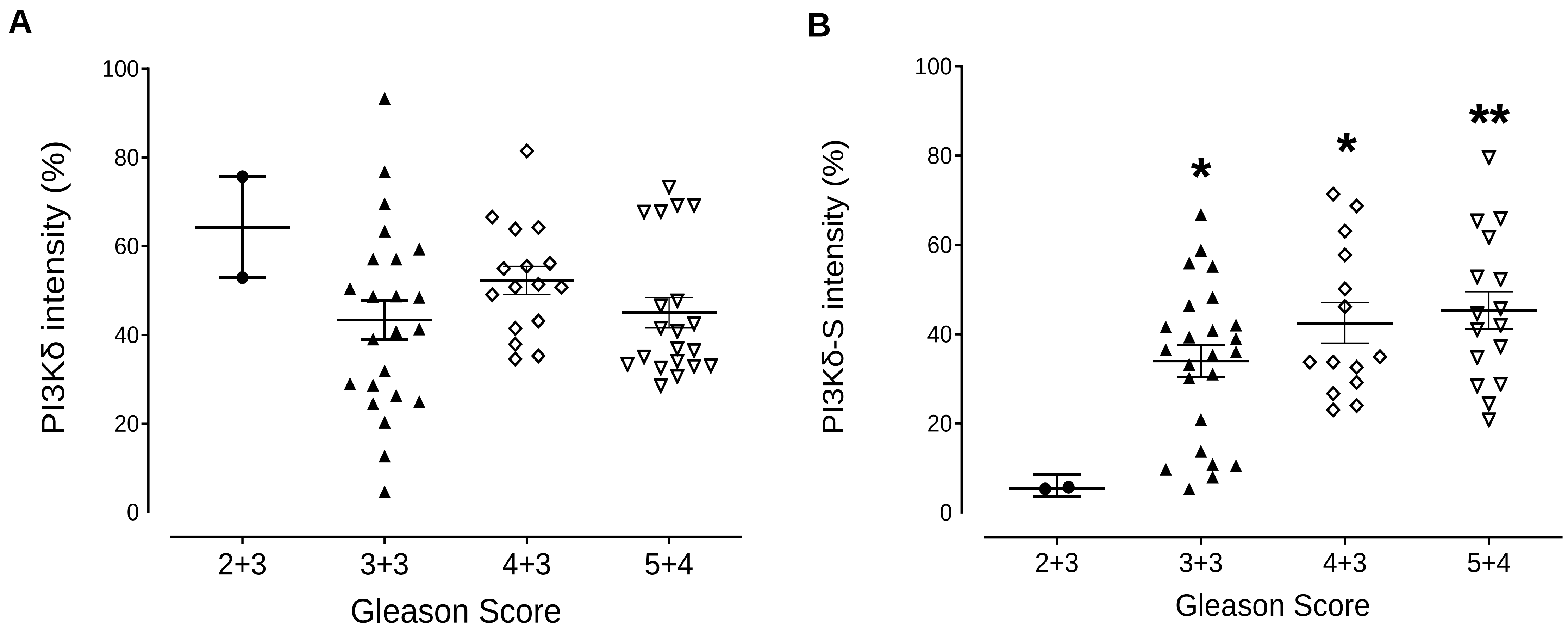

Supplementary Figure S2. Higher PI3Kδ-S levels are correlated to higher Gleason scores. (A) Normalized PI3Kδ and **(B)** PI3Kδ-S intensities in PCa specimens with Gleason scores of 2+3, 3+3, 4+3, and 5+4. The PI3Kδ and PI3Kδ-S intensities were measured based on IHC images. Significantly different PI3Kδ-S intensities (*$p < 0.05$ and **$p < 0.01$, one-way ANOVA with Tukey's post hoc test) between patients with higher Gleason scores (4+3 or 5+4) and low Gleason scores (2+3). Data values were based on mean ± SEM.

Supplementary Figure S3. IHC staining results revealed PI3Kδ-S as a potential precision prognostic biomarkers in endocrine and solid tumors. Representative IHC images of PI3Kδ and PI3Kδ-S levels in different pathological grades of **(A)** prostate cancer, **(B)** breast cancer, **(C)** pancreatic cancer, **(D)** colon cancer, and **(E)** lung cancer specimens. Different Gleason Scores were represented as 2+3, 3+3, 4+4, and 5+4. G1: grade 1; G2: Grade 2; G3: Grade 3. The intensities/scores of PI3Kδ and PI3Kδ-S were labeled on the top-left comers of IHC images.

A. Prostate Cancer

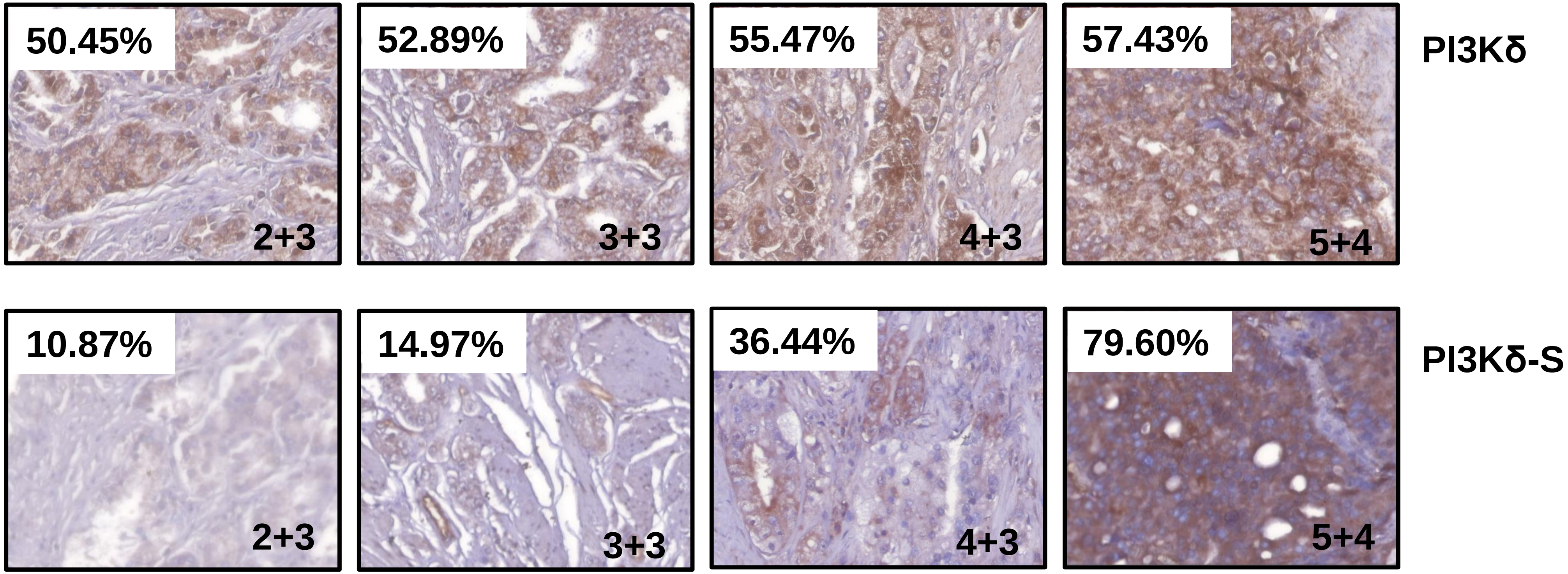

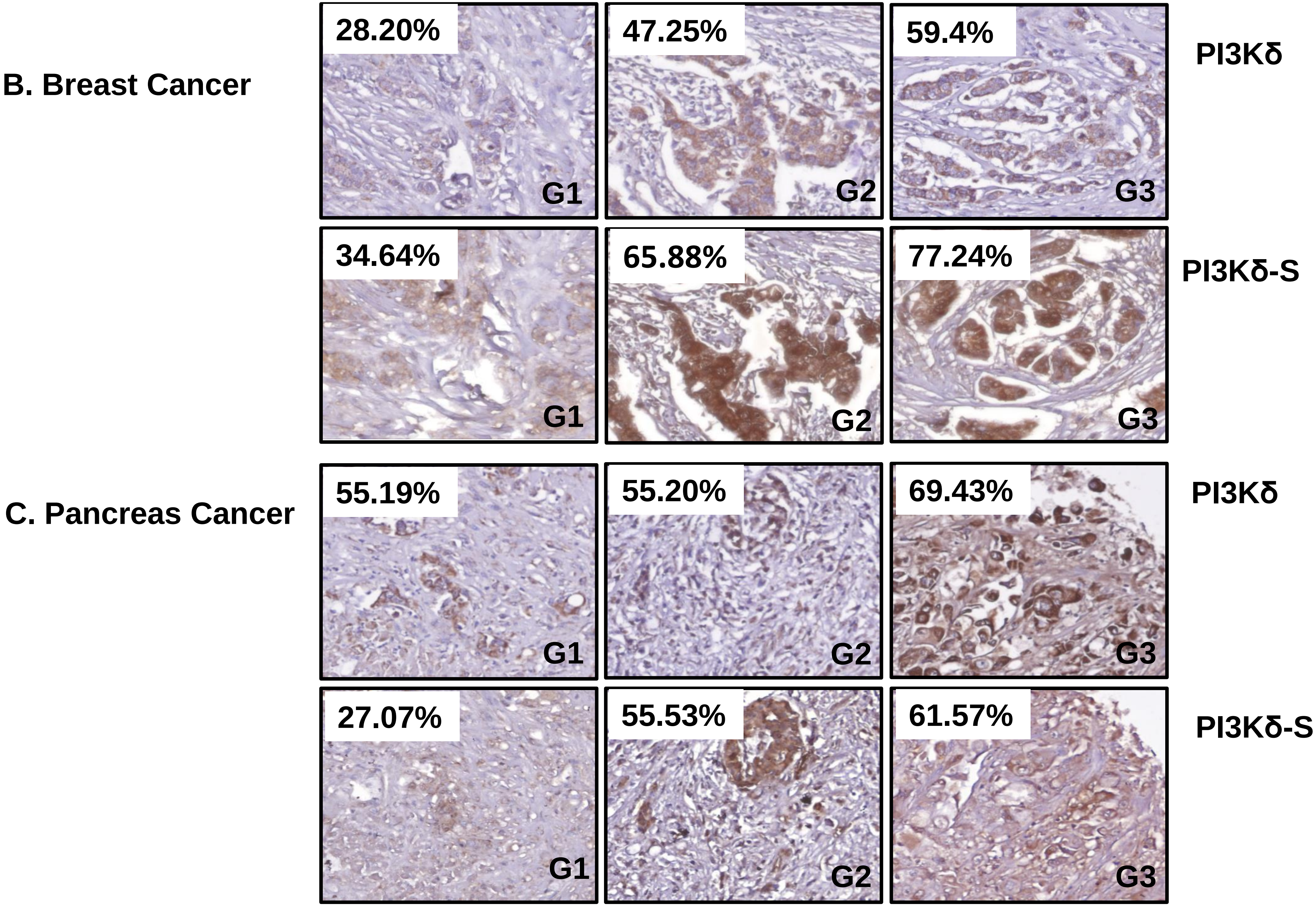
B. Breast Cancer
28.20%
G1
47.25%
G2
59.4%
G3
PI3Kδ
34.64%
G1
65.88%
G2
77.24%
G3
PI3Kδ-S
C. Pancreas Cancer
55.19%
G1
55.20%
G2
69.43%
G3
PI3Kδ
27.07%
G1
55.53%
G2
61.57%
G3
PI3Kδ-S

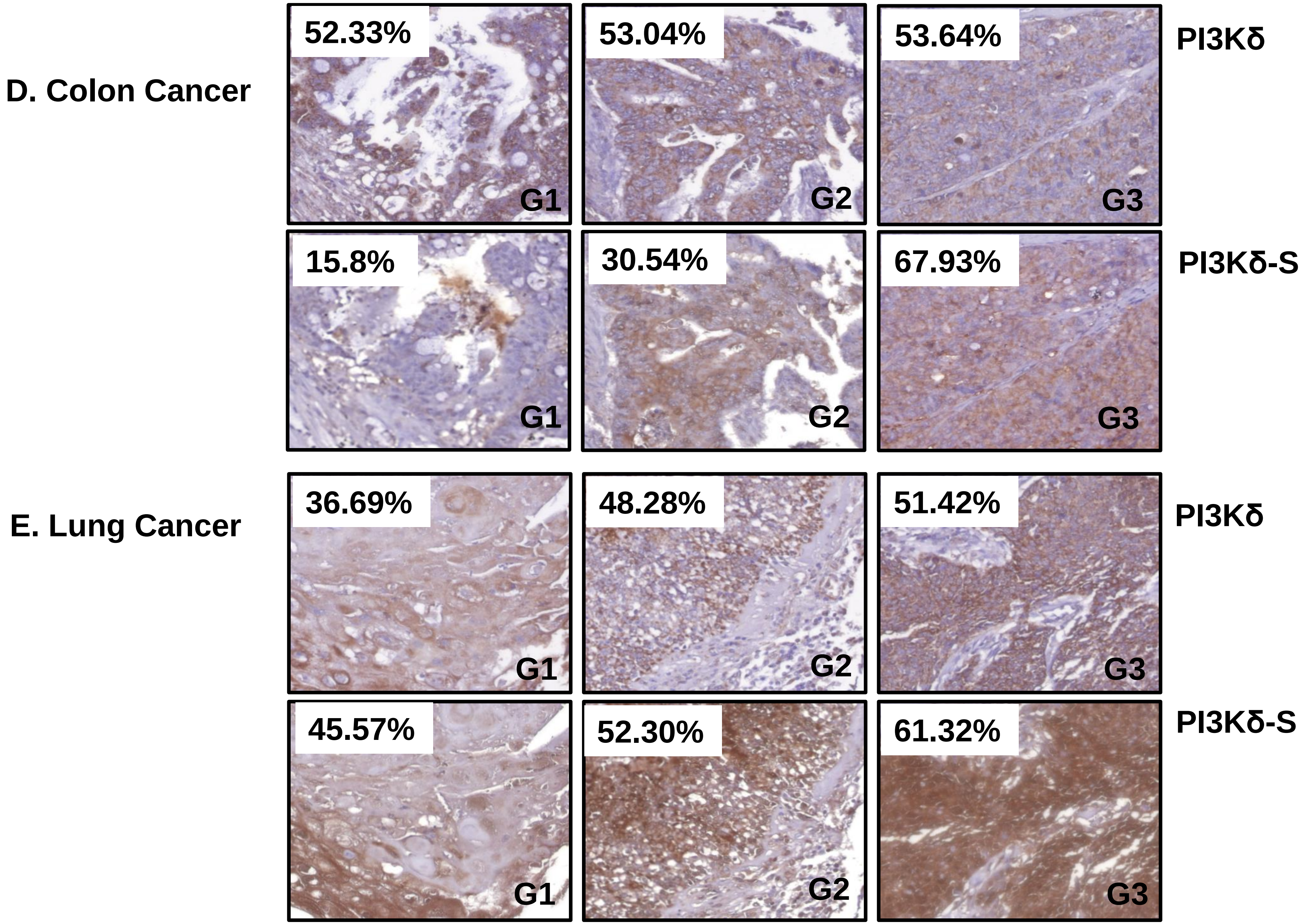

D. Colon Cancer
52.33%
G1
53.04%
G2
53.64%
G3
PI3Kδ
15.8%
G1
30.54%
G2
67.93%
G3
PI3Kδ-S
E. Lung Cancer
36.69%
G1
48.28%
G2
51.42%
G3
PI3Kδ
45.57%
G1
52.30%
G2
61.32%
G3
PI3Kδ-S

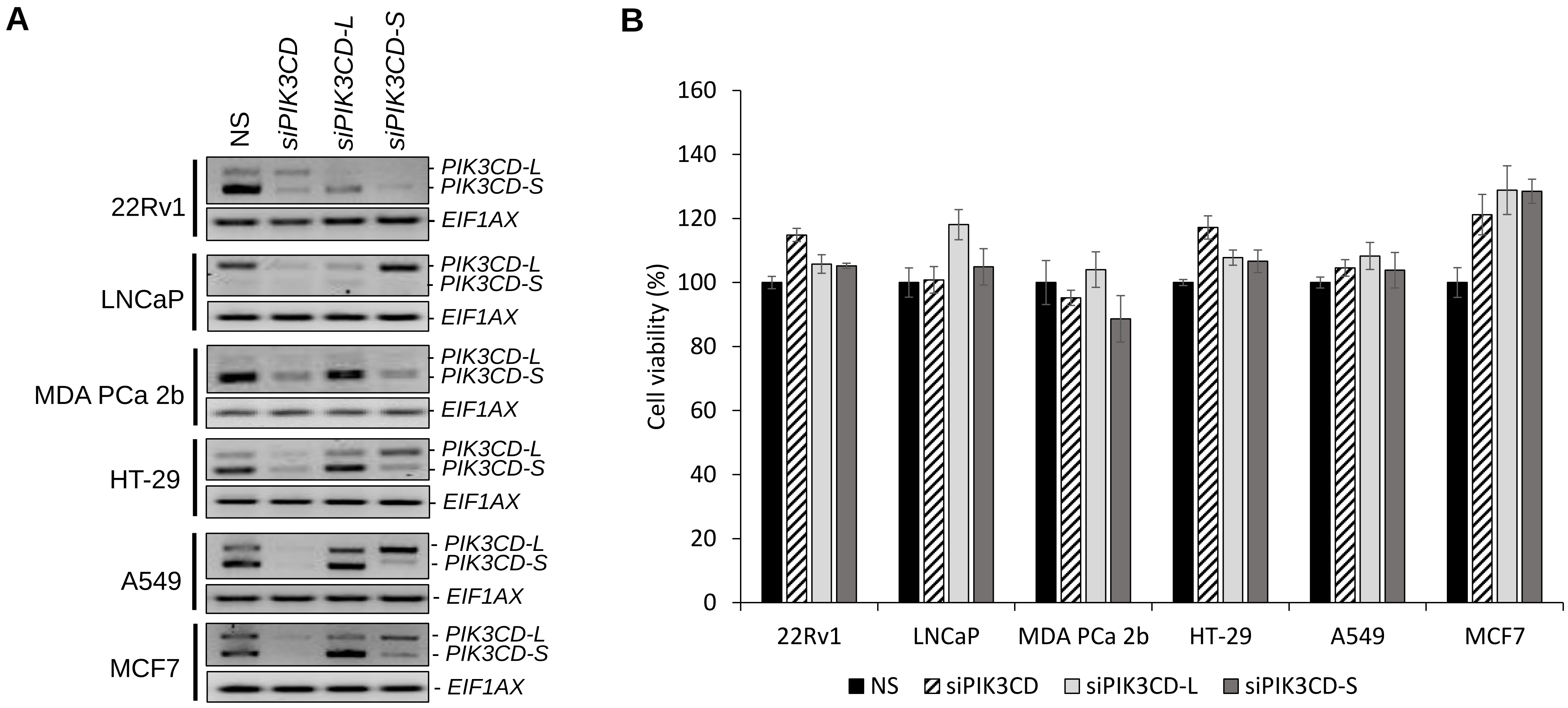

Supplementary Figure S4. SiRNA knockdown of PIK3CD splice variants followed by cell viability assays. (A) RT-PCR assays to verify the efficiencies of siRNA knockdown of total *PIK3CD* splice variants (*siPIK3CD*), *PIK3CD-L* (*siPIK3CD-L*), and *PIK3CD-S* (*siPIK3CD-S*). *EIF1AX* was used as endogenous control for RT-PCR assay. **(B)** Cell viabilities after the cancer cells were transfected with siRNAs for 24 h. The data were presented as mean ± SD, from 3-4 independent experimental repeats.

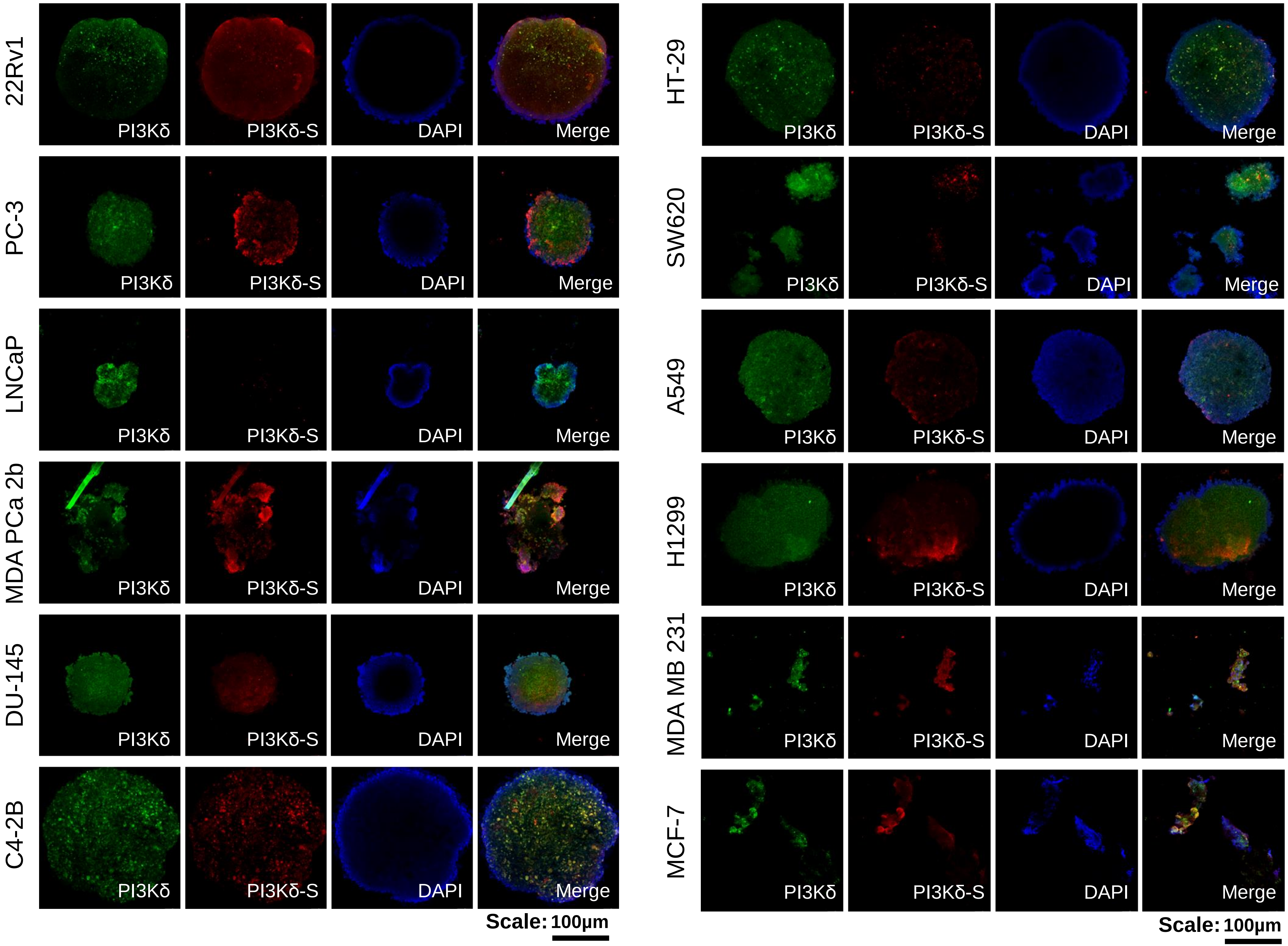

Supplementary Figure S5. Immunofluorescence assays of PI3Kδ and PI3Kδ-S in PCa and endocrine/solid tumors. Immunofluorescence images revealed the expression levels of PI3Kδ (green) and PI3Kδ-S (red) in 22Rv1, PC-3, LNCaP, MDA PCa 2b, DU-145, C4-2B, HT-29, SW620, A549, HT299, MDA MB 231, and MCF-7 spheroids.

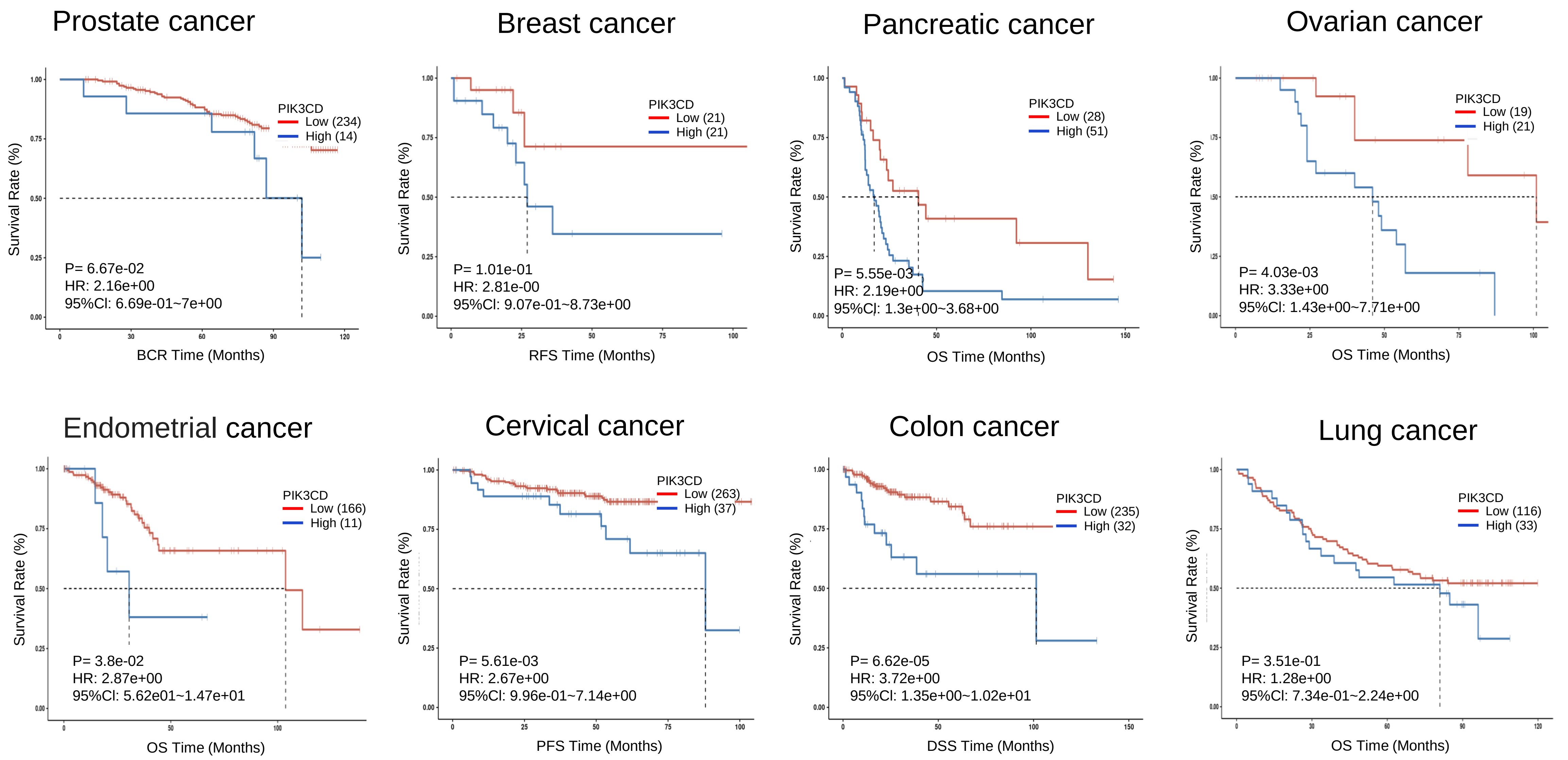

Supplementary Figure S6. Survival curves for endocrine/solid tumor patients expressing high and low levels of PI3Kδ. Significantly lower survival rates were observed in endocrine/solid tumor patients expressing high-level PI3Kδ, vs. low-level PI3Kδ. OS: overall survival; BCR: biochemical relapse; PFS: progression-free survival; RFS: relapse-free survival; DSS: disease-specific survival.
